# Multilocus Phylogeny of Asiatic Striped Squirrels (Sciuridae, *Tamiops*) Reveals Their Evolutionary Relationships and Species Limits

**DOI:** 10.1002/ece3.73099

**Published:** 2026-02-11

**Authors:** Yan Zou, Lange Hui, Wenhao Huang, Rong Ji, Xinyu Tang, Xuming Wang, Shunde Chen, Song Li, Shaoying Liu, Keyi Tang

**Affiliations:** ^1^ College of Life Sciences Sichuan Normal University Chengdu Sichuan China; ^2^ Sichuan Academy of Forestry Chengdu Sichuan China; ^3^ Kunming Natural History Museum of Zoology, Kunming Institute of Zoology Chinese Academy of Sciences Kunming Yunnan China

**Keywords:** mitochondrial genomes, nuclear genes, phylogenetic analysis, *Tamiops*, taxonomic revision

## Abstract

The genus *Tamiops*, classified within the family Sciuridae and the subfamily Callosciurinae, is distributed across East and Southeast Asia. The taxonomic classification of *Tamiops* has historically been contentious, with species diversity often underestimated due to reliance on pelage color variations for species descriptions, which are thought to reflect seasonal changes. To clarify the internal phylogenetic relationships and taxonomic status of the *Tamiops* species, we conducted morphological analyses and constructed multi‐locus phylogenetic trees using a large DNA dataset that encompassed one mitochondrial locus (Cyt‐b) and three nuclear loci (IRBP, PRKCI, RAG1), as well as 20 complete mitochondrial genomes. We identified seven well‐supported monophyletic clades of *Tamiops* based nuclear data evidence. The K2P genetic distances among the seven lineages of *Tamiops* varied from 0.095 to 0.204. Consequently, we elevated one subspecies*—* *T*. *maritimus hainanus* *—*to full species status, having diverged from its common ancestor approximately 6.01 million years ago. Ultimately, we recognized a total of seven valid species within the genus *Tamiops*, which include *T*. *swinhoei* , *T*. *maritimus* , *T*. *hainanus*, *T*. *barbei* , *T*. *roldolphii*, *T*. *minshanica*, and *T*. *mcclellandii* . Divergence time estimation suggested that *Tamiops* began to diversify approximately 13.3 million years ago (Mya). Our findings provide insights into the evolutionary relationships and previously overlooked species diversity within the genus *Tamiops*.

## Graphical abstract

The genus *Tamiops* has long been underestimated in terms of species diversity due to reliance on seasonal fur coloration. This study integrates mitochondrial whole‐genome and nuclear gene data to identify seven monophyletic lineages, elevating *T*. *maritimus hainanus* to independent species status. Furthermore, it infers that divergence within the genus began approximately 13.3 million years ago, revealing its hidden diversity.

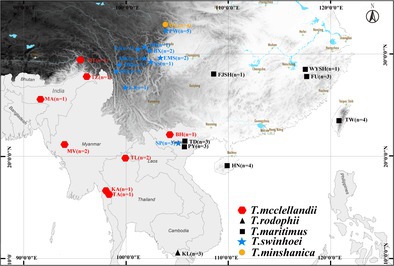

## 1 Introduction

The Asian striped squirrels of the genus *Tamiops* (Rodentia, Sciuridae, Callosciurinae) (J. A. Allen 1906; Wei 2022), encompass six distinct species: *Tamiops mcclellandii* (Horsfield 1840), *Tamiops rodolphii* (M. A. Milne‐Edwards 1867), *Tamiops maritimus* (Bonhote 1900), *Tamiops swinhoei* (M. A. Milne‐Edwards 1874), *Tamiops minshanica* Liu et al. 2022 and *Tamiops barbei* (Blyth 1847). The geographical distribution of these species is primarily concentrated in East and Southeast Asia, with five of the species found in China, while *T*. *rodolphii* is distributed in Vietnam, Cambodia, Thailand, and Laos (Wilson et al. 2016; Liu et al. 2022; Wei 2022; Hinckley et al. 2024).

The five species of the genus *Tamiops* distributed in China encompass 16 subspecies, which are categorized as follows: four subspecies of *T*. *maritimus* , eight subspecies of *T*. *swinhoei* , two subspecies of *T*. *barbei* , and *T*. *mcclellandii* and *T*. *minshanica*, which are not subdivided into subspecies (Wei 2022; Hinckley et al. 2024). However, classifying species and subspecies within the *Tamiops* genus based on pelage coloration has proven challenging, as there is great seasonal variation in pelage coloration, particularly in the temperate species (Moore and Tate 1965; Li et al. 2006; Chang et al. 2011). For instance, *T*. *swinhoei* and *T*. *maritimus* were previously regarded as the same species due to their similar stripe patterns and body size (Corbet and Hill 1992). Therefore, among them, some forms may be tentative and considered synonyms (Liu et al. 2022). In 1874, Milne‐Edwards described *T*. *swinhoei* as a subspecies of *S*. *mcclellandii*, naming it *Sciurus mcclellandii* var. *swinhoei* (M. A. Milne‐Edwards 1874). This taxon was later elevated to a full species by Thomas (1911), who named it *T*. *swinhoei* . Currently, *T*. *swinhoei* is primarily distributed in China, Myanmar, Vietnam, and other regions. It comprises eight subspecies (*T*. *s*. *swinhoei*, *T. s. forresti*, *T. s. russeolus*, *T. s. vestitus*, *T. s. olivaceus*, *T. s. spencei*, *T. s. chingpingensis* and *T*. *s*. *markamensis*), all of which are found in China (Wei 2022). J. A. Allen (1906) described the characteristics of the molariform teeth of *Sciurus vulgaris* which differ from other *Sciurus* species and elevated *Tamiops* to a separate genus containing two species: *T*. *mcclellandii* and *T*. *maritimus* . In G. M. Allen 1940, G. Allen relegated *T*. *monticolus* to synonymy and classified *T*. *maritimus* as a subspecies. Currently, *T*. *maritimus* is primarily distributed in China, Laos, Vietnam, and other regions. It includes four subspecies, with *T*. *m*. *maritimus* and *T*. *m*. *hainanus* found in China. Furthermore, previous studies have indicated that species diversity may have been underestimated, suggesting that eight putative species could be recognized within the genus *Tamiops*. As *T*. *swinhoei* and *T*. *maritimus* are not monophyletic in the phylogenetic tree but instead form *T*. *swinhoei* *—maritimus* complex (Chang et al. 2011; Zhang et al. 2019). Therefore, given the underestimated species diversity within this genus, it is necessary to further assess species diversity and clarify species boundaries within in genus *Tamiops*.

The mitochondrial genomes (mitogenomes), characterized by a high number of intracellular copies, matrilineal inheritance, lack of recombination, small molecular weight, and rapid evolutionary rates, have been widely utilized for studying genetic diversity in populations, molecular phylogeography (Hinckley et al. 2023; Profant et al. 2024; Wang et al. 2024), as well as for phylogenetic analysis and comparative genomics (Ji et al. 2023; Wu et al. 2022). With the advent of next‐generation sequencing (NGS) technology, an increasing number of mitogenomes have been assembled in the Sciuridae family (Hu et al. 2016; Hawkins et al. 2016; İbiş et al. 2022; Matrosova et al. 2023). Currently, the mitogenomes of only three species are available in the NCBI database: *T*. *mcclellandii* , *T*. *barbei* , and *T*. *maritimus* (Cong et al. 2017; Hinckley et al. 2023), *T. minshanica* and *T*. *rodolphii* lack mitogenome data.

In order to improve our understanding on the evolutionary history of these striped squirrels, we incorporated molecular and morphological data for one of *T*. *maritimus* recognized subspecies (*T. m. hainanus*) in Hainan Island and three recognized subspecies of *T*. *swinhoei* (*T. s. clarkei*, *T. s. russeolus*, and *T. s. forresti*) in the mountains of Southwest China. We constructed phylogenetic analysis based on mitogenomes, one mitochondrial and three nuclear loci. Additionally, craniodental and external indicators were measured to conduct morphological analyses. We aimed to (1) re‐evaluate molecular phylogenetic relationship within the genus *Tamiops*, (2) resolve taxonomic uncertainties, especially concerning the taxonomic status of *T. m. hainanus*, and (3) characterize mitogenomes of Asian striped squirrels.

## 2 Materials and Methods

### 2.1 Sample Collection

**FIGURE 1:**
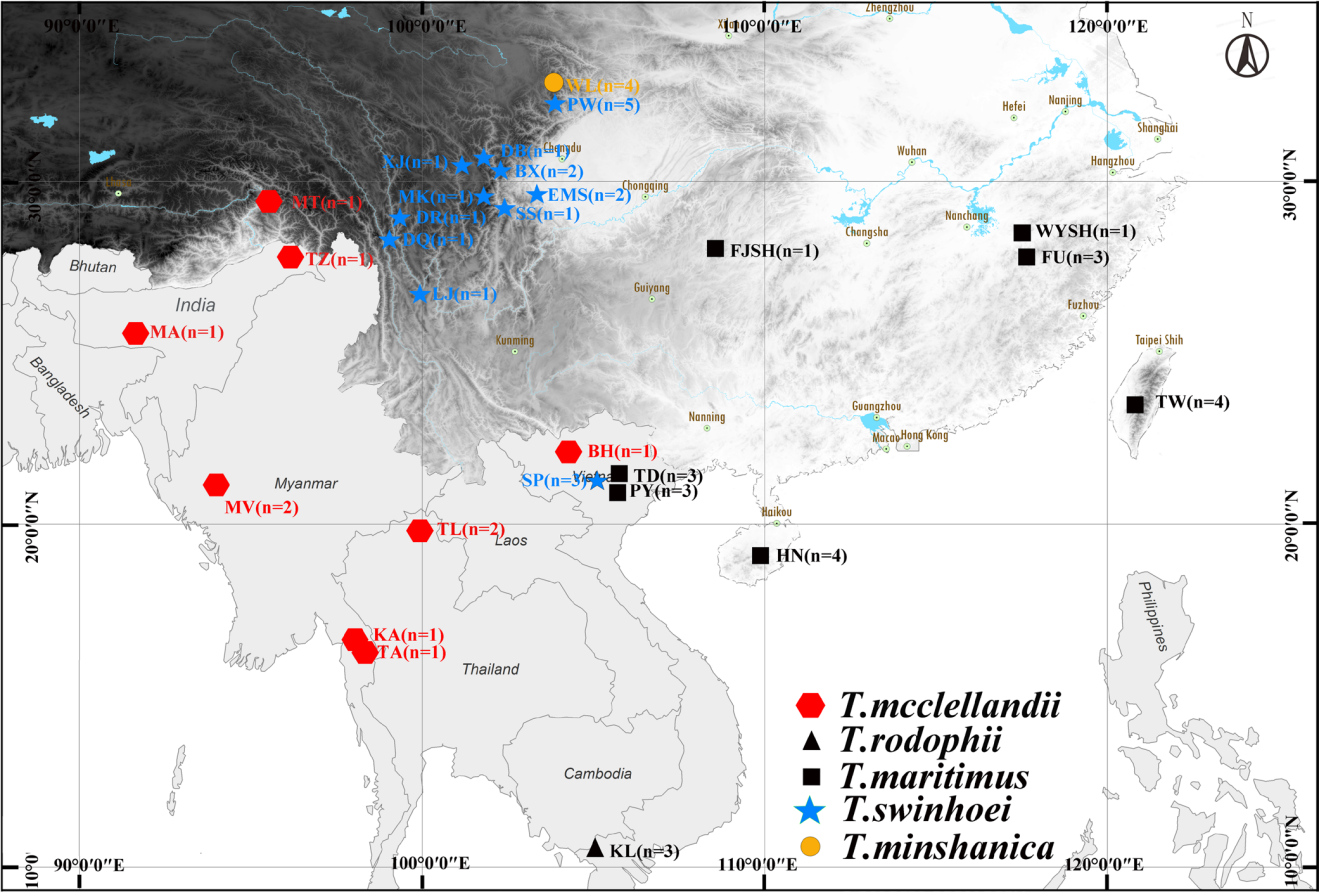
Locality of species within the genus *Tamiops* in this study.

A total of 21 *Tamiops* specimens were collected in China, including 15 individuals of *T*. *swinhoei* . This collection comprises 12 *T*. *s swinhoei* (two of which are from the type locality, Baoxing), one *T*. *s*. *clarkei*, one *T*. *s*. *russeolus*, and one *T*. *s*. *forresti*, all from Sichuan and Yunnan. Additionally, one specimen of *T*. *mcclellandii* was collected from Motuo in Tibet, which is near the type locality. One specimen of *T*. *minshanica* was obtained from Wanglang in Pingwu County (the type locality), Sichuan Province. Furthermore, three specimens of *T*. *maritimus hainanus* were collected from Hainan (the type locality), and one *T*. *maritimus* was found at Fanjingshan Mountain in Guizhou. Detailed specimen information is listed in Table 1 and Figure 1, and Table S1. All liver and muscle tissues of the *Tamiops* species were collected and stored at −80°C at College of Life Sciences, Sichuan Normal University. All dry preserved specimens were deposited in the Sichuan Academy of Forestry Sciences. All samples were collected in accordance with the approval (2024LS020) and guidelines of the Animal Ethics Committee to ensure the ethical treatment of animals at Sichuan Normal University.

**TABLE 1:** A list of the complete mitochondrial genomes of the 20 individuals from the genus *Tamiops* utilized in this study.

Lab No.^a^	Museum No.^b^	Species	Genbank No.	Mitogenome in length	Collection location	References
JJSA442	SAF09282	*Tamiops swinhoei swinhoei*	PQ591910	16,501 bp	Baoxing, Sichuan, China	In this study
CSD6395		*T*. *s*. *swinhoei*	PQ591911	16,501 bp	Emeishan, Sichuan, China	In this study
DR01‐004	SAF14745	*T*. *s*. *clarkei*	PQ591915	16,506 bp	Derong, Sichuan, China	In this study
YN15034	SAF15254	*T*. *s*. *russeolus*	PQ591920	16,506 bp	Deqin, Yunnan, China	In this study
LJ19018	SAF19814	*T*. *s*. *forresti*	PQ591918	16,511 bp	Lijiang, Yunnan, China	In this study
FJSH13049	SAF13049	*T*. *maritimus*	PQ591916	16,519 bp	Tongren, Guizhou, China	In this study
CSD7747	SAF201148	*Tamiops maritimus hainanus*	PQ591913	16,523 bp	Hainan, China	In this study
CSD7748		*T*. *m*. *hainanus*	PQ591914	16,526 bp	Hainan, China	In this study
KDS20001		*T*. *m*. *hainanus*	PQ591917	16,528 bp	Hainan, China	In this study
WL18668	SAF181596	*Tamiops minshanica*	PQ591919	16,559 bp	Wanglang, Sichuan, China	In this study
CSD6956		*Tamiops mcclellandii mcclellandii*	PQ591912	16,523 bp	Motuo, Xizang, China	In this study
		*T*. *m*. *mcclellandii*	OQ160760.1	16,629 bp	Mt Victoria, Chin Hills, Myanmar	Hinckley et al. (2023, 2024)
		*T*. *m*. *mcclellandii*	OQ160761.1	16,629 bp	Mt Victoria, Chin Hills, Myanmar	Hinckley et al. (2023, 2024)
		*T*. *m*. *mcclellandii*	OQ160762.1	16,629 bp	Tezu, Mishmi Hills, Assam, India	Hinckley et al. (2023, 2024)
		*T*. *m*. *mcclellandii*	OQ160759.1	16,629 bp	Mawphlang, Khasi Hills, Assam, India	Hinckley et al. (2023, 2024)
		*Tamiops barbei*	OQ160754.1	16,629 bp	Taok, Tenasserim (Tanintharyi), Myanmar	Hinckley et al. (2023, 2024)
		*T*. *barbei*	OQ160753.1	16,629 bp	Kawkereik, Tenasserim (Tanintharyi), Myanmar	Hinckley et al. (2023, 2024)
		*T*. *b*. *inconstans*	OQ160757.1	16,629 bp	Bao‐Ha, Cac Ba, Lao Cai, Vietnam	Hinckley et al. (2023, 2024)
		*T*. *maritimus*	KP027416.1	16,513 bp	Wuyishan, Jiangxi, China	Xu et al. (2016)
		*T*. *maritimus*	KP708710.1	16,523 bp	Baoting, Hainan, China	Cong et al. (2017)

^a^ Lab No. Field Collection Number.

^b^ Museum No. Forestry Academy Museum Number.

### 2.2 DNA Extraction, PCR Amplifications, and Sequencing

Total genomic DNA was extracted from muscle tissue using the Animal Tissue DNA Isolation Kit (Foregene Co Ltd) following the manufacturer's introductions. Species were initially identified based on morphological characteristics and distribution maps, as described by Smith and Xie (2009). Furthermore, polymerase chain reaction (PCR) was used to amplify *Cytochrome b* (Cyt‐b, 1140 bp) gene sequence for species identification. In addition, PCR reactions were employed to amplify segments of three nuclear genes from 17 individuals: interphotoreceptor retinoid‐binding protein [IRBP, 1016 bp], protein kinase C iota [PRKCI, 436 bp], and recombination‐activating gene 1 [RAG1, 1190 bp]. PCR primers for all four genes referred to previous study (Table 2) (He et al. 2010; Stanhope et al. 1992; Matthee et al. 2004; Steppan et al. 2004). The PCR thermal cycling profile of the Cyt‐b gene was as follows: an initial denaturation at 94°C for 5 min, followed by 38 cycles of 94°C for 40 s, annealing at 49°C for 45 s, and extension at 72°C for 1.5 min. A final extension was performed at 72°C for 10 min. The procedures were consistent across all four genes, with the exception of the annealing temperatures: 53°C for IRBP, 50°C for PRKCI, and 51°C for RAG1. PCR products were analyzed on a 1% agarose gel for quality assessment and subsequently purified for sequencing. The purified PCR products were sent to Tsingke Biotech Co. Ltd for Sanger sequencing in both directions. Sequences were assembled and edited using MEGA (Version 5.0) (Tamura et al. 2011). DNA sequences were aligned using the BLAST tools available in NCBI to identify the species. All Cyt‐b gene and three nuclear gene sequences obtained in this study are available in GenBank (Accession numbers: PQ629529—PQ629586, Table S1).

**TABLE 2:** Primers used for PCR in this study.

Genes	Primers	Sequences	References
CYTB	L14724(hk3)	GGACTTATGACATGAAAAATCATCGTTG	He et al. (2010)
	H15915(hk3)	GATTCCCCATTTCTGGTTTACAAGAC	He et al. (2010)
	HSS(F)	ATGACAAATATCCGCAAAACCCA	This study
	HSS(R)	TCTTCATTTAAGGAGTTTGTTTTCG	This study
IRBP	+IRbP217(f)	ATGGCCAAGGTCCTCTTGGATAACTACTGCTT	Stanhope et al. (1992)
	‐IRbP1531(f)	CGCAGGTCCATGATGAGGTGCTCCGTGTCCTG	Stanhope et al. (1992)
RAG1	S70 (f)	TCCGAGGGAAATTTAAGMTGTT	Steppan et al. (2004)
	S73 (r)	GAGGAAGGTRTTGACACGGATG	Steppan et al. (2004)
PRKCI	Pk1 (f)	AAACAGATCGCATTTATGCAAT	Matthee et al. (2004)
	Pk2 (r)	TGTCTGTACCCAGTCAATATC	Matthee et al. (2004)

### 2.3 Library Preparation and Next‐Generation Sequencing

A total of 0.2 μg of genomic DNA per sample was utilized for DNA library construction. Briefly, the genomic DNA was fragmented using the Covaris LE220R‐plus (Covaris, USA) to a size of 350 bp. The DNA fragments were then end‐polished, A‐tailed, and ligated with the index adapter for subsequent sequencing, followed by further PCR amplification. Library quality was assessed using the Agilent 5400 system (AATI) and quantified through real‐time PCR. The qualified library was subsequently sequenced with 150 bp paired‐end reads on the Illumina NovaSeq 6000 platform (Illumina, San Diego, CA) at Novogene Bioinformatics Technology Co. Ltd (Beijing, China). Clean data were generated after removing adapter sequences, ‘N’‐rich reads, and low‐quality reads (Phred quality < 5). For each library, clean data ranging from 4.32 to 19.36 GB were obtained following the processing of raw data.

### 2.4 Mitogenome Assembly, Annotation, and Sequence Analysis

Adaptor removal and quality trimming was performed with Trimmomatic (Version 0.39) (Bolger et al. 2014). The mitogenomes of 11 individuals from the genus *Tamiops* were assembled and generated using Geneious Prime (Version 9.0.2) software (Kearse et al. 2012) and MitoZ (Version 2.4) (Meng et al. 2019). We used Geneious to generate consensus sequences, with the threshold set to “Highest Quality” and other parameters at their default values and then employed MEGA (Version 5.0) to remove duplicate fragments. The mitogenomes of *T*. *maritimus* (GenBank accession no. KP708710.1) and *T*. *mcclellandii* (GenBank accession no. OQ160759.1), downloaded from NCBI and used as reference templates. The assembled complete mitogenomes were verified and manually revised using MEGA (Version 5.0). The consensus mitogenome sequence was annotated with the MITOS2 Web Server (http://mitos2.bioinf.uni‐leipzig.de/index.py) (Bernt et al. 2013). The basic features of protein‐coding genes (PCGs) were characterized, including their locations, whether they were on the light or heavy strand, and the start and stop codons. The secondary structure of tRNAs were predicted using the tRNAscan‐SE Web Server (Chan et al. 2021). The draft of tRNAs was generated using VARNA (Version 3.93) (Darty et al. 2009). The gene maps of the mitogenomes were created using the CGView server (https://proksee.ca/) (Grant and Stothard 2008). We calculated the nucleotide bias (GC skew and AT skew) using the formulas: “AT‐skew = (A – T)/(A + T)” and “GC‐skew = (G − C)/(G + C)” (Perna and Kocher 1995). All PCGs were aligned using MEGA (Version 5.0) to ensure that each gene could be accurately translated. After removing the termination codon, both codon usage and relative synonymous codon usage (RSCU) were calculated using MEGA (Version 5.0). Non‐synonymous substitutions (Ka) and synonymous substitutions (Ks) of the 13 PCGs were analyzed separately using DnaSP (Version 5.1) (Rozas et al. 2017). The comparison of the Ka/Ks ratio between species was visualized using the “ggplot2” package in R software. The results of the mitochondrial genome analysis are presented in Appendix S1.

### 2.5 Phylogenetic Analysis

We employed maximum likelihood (ML) and Bayesian inference (BI) methods to construct phylogenetic trees. Four genetic datasets were prepared: (1) Cyt‐b, (2) nuDNA (IRBP + PRKCI + RAG1), (3) Cyt‐b + nuDNA, and (4) 13 PCGs from complete mitogenomes. We retrieved nine published complete mitogenomes, 28 Cyt‐b sequences, and 82 nuclear gene sequences of *Tamiops* species from GenBank. Additionally, we downloaded Cyt‐b gene sequences and three nuclear gene sequences from the genera *Callosciurus*, *Sundasciurus*, and *Dremomys* from GenBank to serve as outgroups. We also obtained the complete mitochondrial genome of *Callosciurus erythraeus* (GenBank accession no. NC_025550.1) (Hu et al. 2016) to be used as an outgroup for the mitochondrial phylogenetic tree. Detailed information on mitogenomes and gene sequences for *Tamiops* taxa is presented in Table 1 and Table S1.

All gene sequences were successfully aligned using MAFFT (Version 7.520) (Katoh et al. 2019). The ML tree was constructed utilizing the IQ‐TREE Web Server (Trifinopoulos et al. 2016), with node confidence assessed through 5000 bootstrap replicates. The BI analysis was performed using BEAST (Version 1.7.5) (Drummond et al. 2012). We selected the best‐fit model for different genes using jModelTest (Version 2.1.7) (Darriba et al. 2012) based on the Akaike information criterion (AIC), as detailed in Table S2 and Table S3. Input files were prepared in BAEUti (Version 1.7.4.) and Bayesian gene trees were reconstructed independently for three datasets, with each run consisting of 100 million generations and sampling every 5000 generations. Convergence was examined using Tracer (Version 1.7) (Rambaut et al. 2018), with a minimum threshold for the effective sample size (ESS) exceeding 200. The BI tree was generated with TreeAnnotator (Version 1.6), which determining burn‐in generations and discarded initial samples. Finally, FigTree (Version 1.3.1) was used for tree visualization. Pairwise genetic distances were calculated using MEGA (Version 5.0) with the Kimura‐2 parametric model.

### 2.6 Estimating Dates of Divergence

Divergence times were estimated using BEAST v.1.7. based on the Cyt‐b dataset. Two fossil calibration points were selected, as referenced by Hinckley et al. (2023). The estimated divergence between the genera *Callosciurus* and *Sundasciurus* occurred approximately 14 Mya, based on the fossil of a *Callosciurus* species (specimen number YGSP 21682) (Flynn 2003). A second fossil calibration point was established at 18.7 Mya, based on paleomagnetic correlation measurements of stemmed fossils of *Tamiops* (specimen numbers Z2438, Z2439, Z2440, Z2441) from the Z122 Vihowa Formation (Flynn 2003). These fossils represent the oldest known records of the *Callosciurus*, *Sundasciurus*, *Tamiops*, and *Dremomys* clades (Hinckley et al. 2023). All fossil age constraints were established using hard minimum and soft maximum boundaries, and they are considered to be lognormally distributed (Parham et al. 2012; Hinckley et al. 2023). We used jModelTest v2.1.3 (Darriba et al. 2012) to determine the optimal evolutionary model. The birth‐death model was employed as the tree prior, and the Relaxed Uncorrelated Lognormal Clock model was used as the clock model prior using BEAUti v1.6.1. Each analysis was conducted for 100 million generations, with sampling conducted every 5000 generations.

### 2.7 Morphological Analyses

A total of 160 complete specimens of intact adult individuals were obtained and measured using a digital caliper calibrated to the nearest hundredth of a millimeter (0.01 mm), as previously described (Table S4; Liu et al. 2022). The morphological measurement indicators for 66 specimens were sourced from Liu et al. (2022) and Hinckley et al. (2023). Prior to conducting principal component analysis (PCA), we performed the Kaiser‐Meyer‐Olkin (KMO) test and Bartlett's test of sphericity. The KMO test yielded a value of 0.882, and the *p*‐value from Bartlett's test of sphericity was less than 0.01, indicating that the morphological data matrix is suitable for PCA. Three external measurements were taken: head‐body length (HBL), tail length (TL), and hind foot length (HFL). For craniomandibular delineation, six variables were measured using a digital caliper calibrated to the nearest hundredth of a millimeter (0.01 mm), including greatest length of skull (GLS), palatal length (PL), zygomatic breadth (ZOB), interorbital breadth (IOB), length of the upper diastema (LUD), and length of the maxillary toothrow (LMXTR). We conducted PCA based on the data matrix to identify morphometric variation among *T*. *swinhoei* (*n* = 29), *T*. *maritimus* (*n* = 35), *T*. *mcclellandii* (*n* = 20), *T*. *rodolphii* (*n* = 5), *T*. *barbei* (*n* = 47), *T*. *minshanica* (*n* = 4), *T*. *hainanus* (*n* = 17), and *T*. *forresti* (*n* = 3) using SPSS v26.0. Missing data were imputed based on species averages, and a scatter plot was generated using SPSS v26.0.

## 3 Results

### 3.1 Phylogenetic Analysis

**FIGURE 2:**
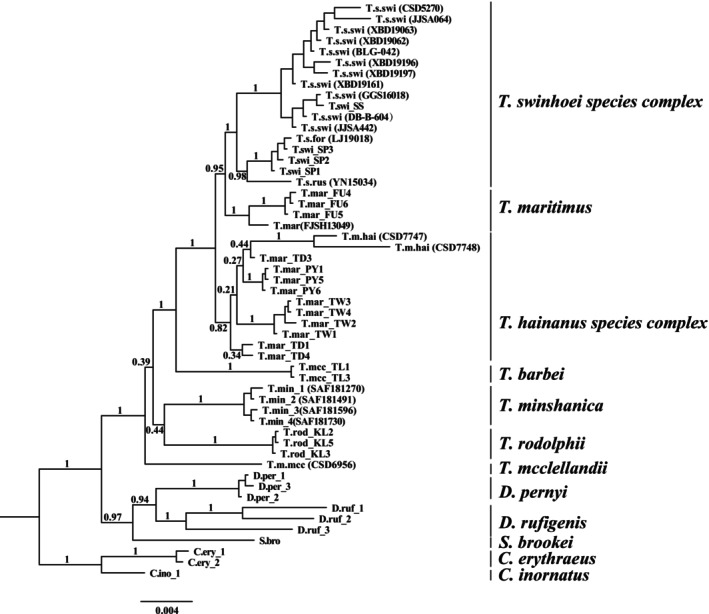
The Bayesian Inference phylogenetic trees of the genus *Tamiops* constructed using nuDNA loci. The numbers above the branches indicate Bayesian posterior probabilities.

**FIGURE 3:**
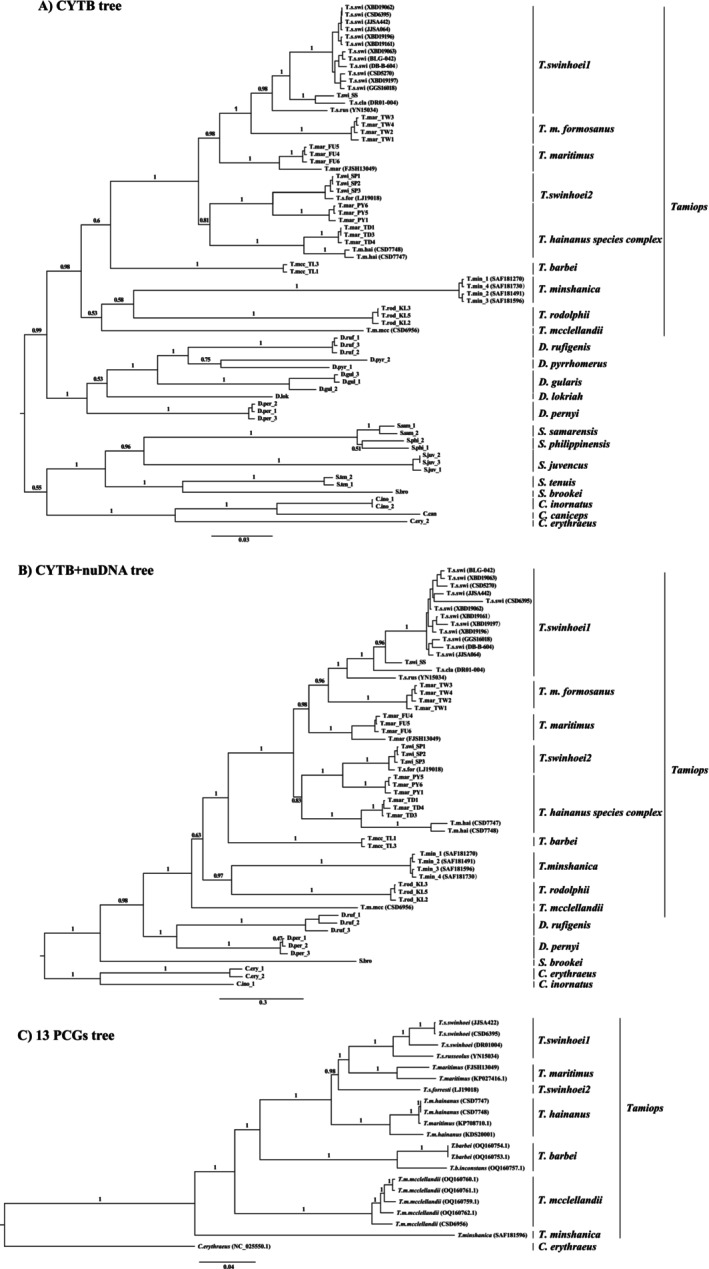
The Bayesian Inference phylogenetic trees of the genus *Tamiops* constructed using genetic markers: (A) Cyt‐b tree, (B) Cyt‐b + nuDNA tree, and (C) 13 PCGs tree. The numbers above the branches indicate Bayesian posterior probabilities.

The phylogenetic relationships within the genus *Tamiops* were inferred by using BI and ML methods based on the nuDNA (Figure 2), Cyt‐b (Figure 3A), Cyt‐b + nuDNA (Figure 3B), 13 PCGs of mitogenomes (Figure 3C). The nuDNA tree recovered seven major clades within the genus *Tamiops* (Figure 2). Among these, *T*. *swinhoei* *–maritimus* complex is divided into three clades, comprising the *T*. *swinhoei* species complex, the *T*. *hainanus* species complex, *T*. *maritimus* , *T*. *barbei*, *T. minshanica*, *T*. *rodolphii* , and *T*. *mcclellandii* (Figure 2). *T*. *m*. *hainanus* is deeply diverged from the *T*. *m*. *maritimus* samples from Fujian, forming a distinct clade. The maximum likelihood and Bayesian phylogenetic reconstructions recovered similar topologies (Figure 3, Figure S1), The mitochondrial and concatenated mitochondrial‐nuclear gene trees also shared a similar topology (Figure 3), however, some differences were observed compared to the nuclear gene tree, reflecting cyto‐nuclear discordance. The *T*. *swinhoei* *–maritimus* complex was divided into five well‐supported subclades. *T*. *swinhoei* has been divided into two clades. The first clade ( *T*. *swinhoei* 1) includes *T*. *s*. *swinhoei* and *T*. *s*. *clarkei* from Sichuan, as well as *T*. *s*. *russeolus* from Yunnan. The second clade ( *T*. *swinhoei* 2) comprises one specimen of *T*. *s*. *forresti* in Lijiang (Yunnan), three *Tamiops* specimens from Vietnam (Figure 3, Figure S1). *T. m. formosanus* from Taiwan, which is sister to *T*. *swinhoei* in the Cytb and Cytb+nDNA trees, but it is nested within *T. hainanus* complex in the nDNA tree. Three specimens of *T*. *m*. *maritimus* from Fujian and one from Fanjinshan Mountain (Guizhou) cluster into a distinct clade. The SP population of the *T*. *swinhoei* 2 clade forms a sister clade to the PY population of *T. hainanus* complex. Five specimens of *T*. *m*. *hainanus* from Hainan form a well‐supported clade, which may deserve full species status and is designated as *T*. *hainanus* (Figure 3). One specimen of *T*. *m*. *mcclellandii* from Medog County forms a distinct clade, while two specimens of *T*. *barbei* from Thailand form another clade. *T*. *minshancia* exhibits a sister relationship to *T*. *roldolphii* based on mitochondrial gene sequences and concatenated datasets of Cyt‐b and three nuclear genes.

We also obtained 11 complete mitogenomes of *Tamiops* species, and combined with nine complete mitogenomes from GenBank, excluding *T*. *rodolphii* , to determine the phylogenetic relationships in the genus *Tamiops*. Phylogenetic analysis of 21 complete mitogenomes, including *C*. *erythraeus* as outgroup, revealed a well‐structured phylogenetic tree (Figure 3C), which was similar to the Cyt‐b and mitochondrial‐nuclear combined gene tree (Figure 3A,B). All lineages resulted in very similar topologies to both mitochondrial and concatenated datasets, exhibiting strong support values (bootstrap support > 98%).

### 3.2 Genetic Distance

We calculated Kimura‐2‐parameter (K2P) distances of the Cyt‐b gene across the seven clades (Table 3). All pairwise K2P distances between the seven clades ranged from 0.095 ( *T*. *swinhoei* vs. *T*. *maritimus* ) to 0.204 ( *T*. *mcclellandii* vs. *T*. *minshanica*). Clearly, all K2P distances were greater than 0.1, except for *T*. *swinhoei* vs. *T*. *maritimus* (0.095), which supports the classification of these seven clades as independent species.

**TABLE 3:** The K2P genetic distance of seven clades derived from phylogenetic trees.

	*T*. *swinhoei*	*T*. *maritimus*	*T*. *mcclellandii*	*T*. *rodolphii*	*T*. *minshanica*	*T*. *barbei*	*T*. *hainanus*
*T*. *swinhoei*							
*T*. *maritimus*	0.095						
*T*. *mcclellandii*	0.171	0.170					
*T*. *rodolphii*	0.173	0.160	0.196				
*T*. *minshanica*	0.182	0.179	0.204	0.188			
*T*. *barbei*	0.147	0.155	0.153	0.164	0.163		
*T*. *hainanus*	0.115	0.119	0.152	0.176	0.178	0.153	

### 3.3 Molecular Dating

**FIGURE 4:**
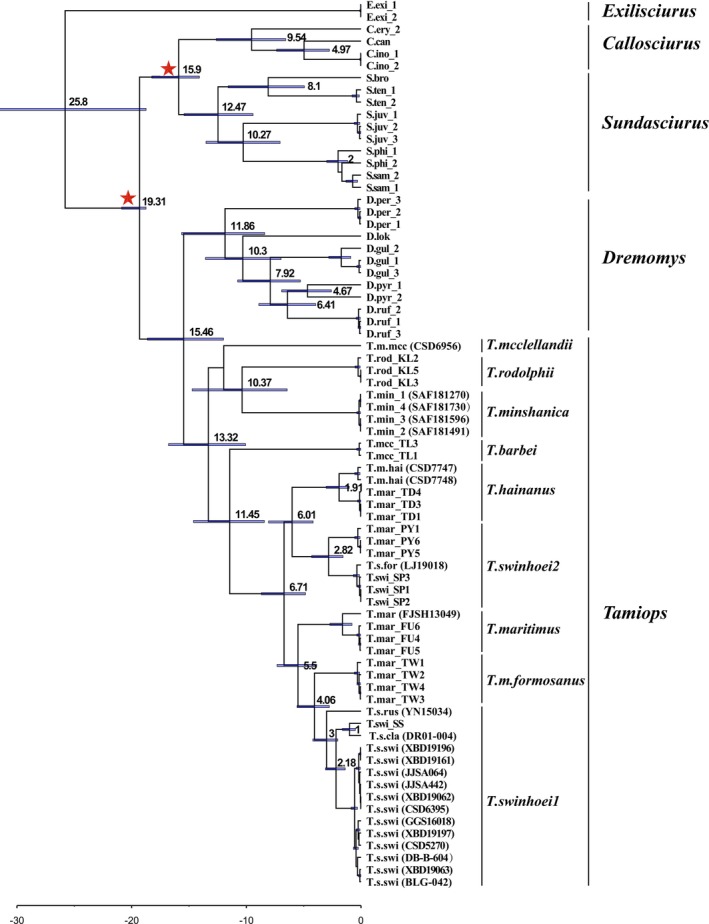
The time‐calibrated phylogenetic tree of the *Tamiops* species is estimated based on the Cyt‐b dataset. The time scale is measured in millions of years ago. The node bars represent the 95% HPD intervals of the divergence time estimates. The red five‐pointed stars indicate the nodes with calibrated dates.

Divergence time estimates indicate that the common ancestor of the genus *Tamiops* dates back to the Early Oligocene, at 13.32 Mya (95% Confidence Interval (CI) = 10.07 ~ 16.78 Mya) (Figure 4). The estimated time for the split between *T*. *rodolphii* and *T*. *minshanica* is 10.37 Mya (95% CI = 6.44 ~ 14.72 Mya). The *T*. *swinhoei* *–maritimus* complex diverged into two major lineages approximately 6.71 Mya (95% CI = 4.84 ~ 8.69 Mya). One of the branches diverged into *T*. *hainanus* and *T*. *swinhoei* 2 6.01 Mya (95% CI = 4.18 ~ 8.07 Mya). *T*. *maritimus* diverged from the *T*. *swinhoei* + *T*. *m*. *formosanus* clade 5.5 Mya (95% CI = 3.87 ~ 7.32 Mya), while *T*. *swinhoei* and *T*. *m*. *formosanus* split 4.06 Mya (95% CI = 2.03 ~ 4.2 Mya).

### 3.4 Morphological Analyses

**FIGURE 5:**
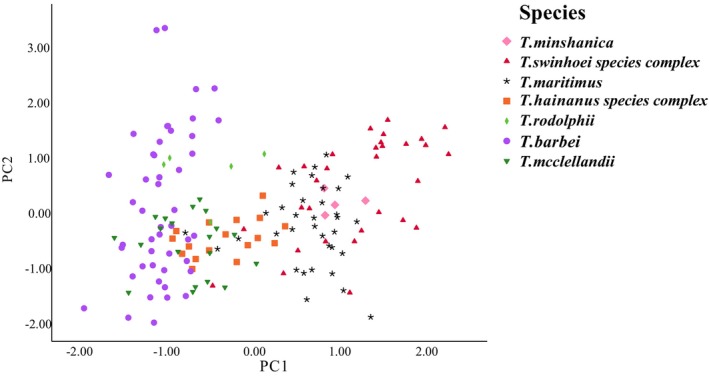
Scatterplot of principal component analysis of morphometric measurements of eight species of the genus *Tamiops*.

The PCA of morphological measurements produced two axes with eigenvalue (5.624 and 1.203), which accounted for 62.49% and 13.36% of the total variance, respectively (Table S5). The first principal component (PC1) exhibited high positive loadings for all variables, with GLS showing the highest loading at 0.969. The second principal component (PC2) demonstrated high positive loadings for all variables except for GLS, ZOB, LUD, and PL. Notably, TL is highly positively correlated with PC2, featuring a factor loading of 0.861. The species generally exhibited a tendency to segregate from each other in a scatter plot (Figure 5). For example, *T*. *swinhoei* and *T*. *barbei* could be clearly distinguished. Although the morphometric indices clearly distinguished *T. hainanus* from the other four congeners ( *T*. *swinhoei* , *T*. *barbei* , *T. minshanica*, and *T*. *rodolphii* ). Specimens of *T*. *mcclellandii* , *T*. *maritimus* , and *T*. *hainanus* overlapped at the center of the scatterplot, suggesting that they have relatively similar body proportions and skull characteristics.

## 4 Discussion

The current taxonomy of the genus *Tamiops* might not adequately reflect its evolutionary history and likely underestimates species diversity. To better resolve the phylogenetic relationships within the *Tamiops* genus, we used Cyt‐b, mitogenomes, and three nuclear markers to re‐examine the taxonomy of this genus. The phylogenetic analysis results showed that seven distinct lineages were recovered in the nuDNA trees (Figure 2).

Previous studies indicated that the *T*. *swinhoei* *–maritimus* complex was not reciprocally monophyletic (Chang et al. 2011; Liu et al. 2022). Chang et al. (2011) recommended that all six populations be classified as distinct species in the *T*. *swinhoei* *–maritimus* complex. In our study, we propose that five species can be recognized from the *T*. *swinhoei* *–maritimus* complex (Figure 3A,B). Specifically, population PY of *T*. *maritimus* form a sister clade to the SP population of *T*. *swinhoei* , and the subspecies *T*. *swinhoei* *forresti* from Lijiang, Yunnan Province, is recognized as a distinct clade (Figure 3A,B). Populations TW and FU of *T*. *maritimus* formed two well‐supported monophyletic groups in both Cyt‐b and mtDNA + nuDNA trees (Figure 3A,B). And population TW of *T*. *maritimus* is generally distributed at elevations between 1500 and 2500 m, which is inconsistent with the general elevation distribution of *T*. *maritimus* (Corbet and Hill 1992). Moreover, the karyotype of population TW (2*n* = 38) significantly differs from that of population FU (2*n* = 42) (Chang et al. 2011). Bonhote (1900) noted that population TW exhibited a darker coloration and overall tone compared to population FU. Bonhote regarded the TW population as a subspecies, *Sciurus mcclellandi formosanus* (Bonhote 1900), which is considered a synonym of *T*. *m*. *maritimus* (Wilson et al. 2016; Wei 2022). The samples from the Hainan, Taiwan, PY, and TD Populations of *T*. *m*. *hainanus* separate from the those from Fujian of *T*. *m*. *maritimus*, suggesting that the taxonomic status of *T*. *m*. *hainanus* should be elevated to full species rank as *T*. *hainanus*, in conjunction with other genetic and morphological evidence.

The subspecies *Tamiops macclellandi hainanus* was first described by J. A. Allen (1906), who noted that its distinctive blackish stripe on the cheeks that extends to the back of the auricle, as well as a darker reddish‐brown stripe on the outer dorsal surface (Figure S2). These features are inconsistent with those of *T*. *m maritimus*. A previous study also indicated that the body size of *T*. *m*. *hainanus* is intermediate between *T*. *maritimus* and *T*. *swinhoei* , suggesting that it might warrant elevation to species status, a notion supported by the well‐resolved monophyletic clade identified in our study (Figure 3A,B) (Wang 2003). The two samples from Hainan and the population TD of *T*. *maritimus* formed two subclades, which were tentatively assigned to the species *Tamiops hainanus*. We also compared the skull sections of *T*. *hainanus* and *T*. *maritimus* , the interspecific differences were not very distinct (Figure S3 and Table S4). *T*. *maritimus* (top row, A1–A4) has a relatively wider skull with a flatter cranial roof (Figure S3). Its rostrum is shorter, and the teeth are robust. The mandible is sturdier, with a broader junction to the cranium. In contrast, *T. hainanus* (bottom row, A1–A4) exhibits a more elongated skull with a distinct cranial crest (Table S4). The eye sockets are relatively smaller and positioned more posteriorly. The mandible is narrower, and the junction with the cranium is more constricted. Our results also support *Tamiops barbei* as a distinct lineage, consistent with Hinckley et al. (2024) assertion to revalidate the specific status of *Tamiops barbei* (Blyth 1847). *T*. *barbei* is differentiated from its parapatric relative, *T*. *mcclellandii* , by its more colorful ventral surface and paler‐tipped tail hairs. In the turnover area between these species, located in western and central Indochina, *T*. *barbei* exhibits a distinctly striped appearance and a longer tail (Hinckley et al. 2024).

Genetic distances in Cyt‐b sequences among the subspecies ranged from 9.5% to 20.4% (Table 3), indicating that these subspecies may merit species status. A previously documented mitochondrial sequence from Mount Wuyi, classified as *T*. *swinhoei* , was re‐evaluated; however, phylogenetic analyses indicate that this specimen is now grouped with *T*. *maritimus* from Fanjingshan Mountain across all phylogenetic trees (Figure 3 and Figure S1). Additionally, considering that Fujian is the type locality for *T*. *maritimus* and that there is no known distribution of *T*. *swinhoei* in that region (Wei 2022), the mitochondrial sequence of *Tamiops* species from Mount Wuyi, previously published on GenBank, should be attributed to *T*. *maritimus* .

Divergence time estimates indicate that *Tamiops* species underwent diversification into multiple lineages during the middle to late Miocene, with an estimated divergence time of approximately 6.71 Mya (95% CI: 4.84 ~ 8.69 Mya) (Figure 4). This period corresponds to the rapid uplift of the Himalayas, which occurred between 10.9 and 7.5 Mya (Amano and Taira 1992; Molnar et al. 2010). In addition, our results indicate that the *T*. *swinhoei* *–maritimus* complex initially diverged into two distinct lineages between 6.71 ~ 3.0 Mya, subsequently diversifying into five lineages. A previous study by Chang et al. (2011) suggested that this complex diverged into two lineages between 5.8 ~ 5.1 Mya, followed by the emergence of four lineages. The inconsistency between the two studies may be attributed to differences in fossil calibrations and the number of samples analyzed.

*T. hainanus* is typically yellowish‐brown, with five black stripes running along the back and sides. The two outer stripes are lighter in color, extending from the nose to the tail. The face features distinct dark eye rings. The inner ears are pale yellow, while the outer ears are covered with soft black fur, ending in pure white tips that form prominent white ear tufts. The underbelly and inner limbs are yellowish‐white, with darker fur at the base. The upper tail exhibits a blend of black and yellowish‐white, with darker fur at the base and yellowish‐white tips, whereas the underside displays a broad reddish‐yellow median stripe edged in black (Figure S2). *Tamiops maritimus* is the smallest species within the genus *Tamiops*, characterized by its small body size. The head is olive‐colored with a few patches of yellow‐brown. The dorsal fur is olive‐gray, while the ventral fur is light yellow. The sides of the body feature short, narrow stripes that are dark brownish‐white. The two central stripes are relatively indistinct, whereas the outer pair is more distinct but less prominent than those found in striped squirrels. The grayish‐white stripes around the eyes do not connect with the other stripes on the back. The back of the ears is black, with a tuft of white hair at the ear tips. The tail displays two black rings separated by a reddish‐yellow ring (Liu and Wu 2019).

### 4.1 Conclusions

In this study, we elucidated the phylogenetic relationships among *Tamiops* species using mitochondrial and nuclear DNA datasets, highlighting previously unrecognized species diversity within the genus. Phylogenetic and morphological analyses suggest that *T*. *m*. *hainanus* may warrant recognition as a distinct species. Consequently, we recognize a total of seven valid species within the genus *Tamiops*: *T*. *swinhoei* , *T*. *maritimus* , *T*. *hainanus*, *T*. *barbei* , *T*. *roldolphii*, *T*. *minshanica*, and *T*. *mcclellandii* . Extensive sampling and taxonomic studies are essential to uncover the true species diversity and classification within the genus *Tamiops*.

## Author Contributions

**Yan Zou:** formal analysis (equal), visualization (equal), writing – original draft (equal). **Lange Hui:** visualization (equal). **Wenhao Huang:** visualization (equal). **Xinyu Tang:** visualization (supporting). **Rong Ji:** software (equal). **Xuming Wang:** visualization (equal). **Shunde Chen:** data curation (equal), resources (equal). **Song Li:** data curation (equal). **Shaoying Liu:** conceptualization (equal), data curation (equal), methodology (equal), resources (equal), writing – review and editing (equal). **Keyi Tang:** conceptualization (equal), data curation (equal), funding acquisition (equal), investigation (equal), project administration (equal), writing – review and editing (equal).

## Funding

This work was supported by Innovation and Entrepreneurship Training Program for Students of Sichuan Normal University, 202410636147. National Natural Science Foundation of China, 32001223.

## Conflicts of Interest

The authors declare no conflicts of interest.

## Supporting information


**Data S1:** ece373099‐sup‐0001‐DataS1.zip.
**Appendix S1:**. Results and discussion of comparative mitochondrial genomes analysis in this study.
**Figure S1:** Maximum likelihood phylogenetic trees of the genus *Tamiops* constructed using various datasets. *A*. *maximum* likelihood phylogenetic tree based on the Cyt‐b dataset; B. Maximum likelihood phylogenetic tree based on the Cyt‐b + nuDNA datasets; *C*. *maximum* likelihood phylogenetic trees based on nuDNA dataset; *D*. *maximum* likelihood phylogenetic trees based on 13PCGs dataset.
**Figure S2:** Photos of the pelage of *Tamiops hainanus* specimens.
**Figure S3:** Comparative Cranial and Mandibular Characteristics of *Tamiops maritimus* and *Tamiops hainanus*. The upper section of the figure, labeled A1–A4, depicts the ventral, dorsal, and lateral views, as well as the mandibular structure of *T*. *maritimus* , respectively. The lower section, also labeled A1–A4, shows the ventral, dorsal, and lateral views, along with the mandibular structure of *T*. *hainanus*, respectively.
**Figure S4:** Circular maps of the mitogenomes of *T*. *swinhoei* (A), *T*. *maritimus* (B), *T*. *mcclellandii* (C), and *T*. *minshanica* (D). Orange blocks represent rRNAs genes, green blocks indicate tRNAs genes, blue blocks denote PCGs, and brownish blocks illustrate the control region and origin of replication.
**Figure S5:** Nucleotide composition of various mitogenome datasets. Hierarchical clustering of *Tamiops* species (*y*‐axis) based on nucleotide content (A) and skewness (B).
**Figure S6:** Relative synonymous codon usage (RSCU) of mitochondrial PCGs in four species of the genus *Tamiops*. The proportion of each amino acid used in the construction of the 13 PCGs is displayed at the top of the bar graph. From left to right, the species represented are *T*. *swinhoei* , *T*. *maritimus* , *T*. *mcclellandii* , and *T*. *minshanica*.
**Figure S7:** Secondary structures from the 22 tRNAs genes of the genus *Tamiops*. The structures of tRNAs genes are presented in the following order: (A) *T*. *swinhoei* , (B) *T*. *maririmus*, (C) *T*. *mcclellandii* , and (D) *T*. *minshanica*.
**Figure S8:** The pairwise nonsynonymous and synonymous ratio (Ka/Ks) of 13 PCGs across six species within the genus *Tamiops*.
**Table S1:** GenBank accession numbers for the DNA sequences used in the phylogenetic analyses of this study.
**Table S2:** Sequence lengths of each gene and the best substitution models employed to reconstruct the phylogenetic tree.
**Table S3:** Sequence lengths of 13 PCGs and the best substitution model employed for reconstructing the phylogenetic tree.
**Table S4:** External and skull measurements of new additions to species within the genus *Tamiops*.
**Table S5:** Factor loadings and percentage of variance explained for principal component analysis on *Tamiops* species.
**Table S6:** General features of the mitochondrial genome of the four species within the genus *Tamiops*, left to right: *T*. *swinhoei* , *T*. *maritimus* , *T*. *mcclellandii* , and *T*. *minshanica*, respectively.

## Data Availability

Data Availability Statement: The newly generated DNA sequences in this study are available in NCBI GenBank (GenBank accession numbers: PQ629529‐PQ629586 for Cyt‐b, IRBP, RAG1, PRKCI; PQ591910‐PQ591920 for mitogenomes).
