# Supplementary material for: Multilocus Phylogeny of Asiatic Striped Squirrels (Sciuridae, Tamiops) Reveals Their Evolutionary Relationships and Species Limits
Source: Ecol Evol. 2026 Feb 11;16(2):e73099. doi: 10.1002/ece3.73099 (PMC12893811; doi:10.1002/ece3.73099)
Supplement: Supplementary file 1 — Data S1: ece373099‐sup‐0001‐DataS1.zip. Appendix S1:. Results and discussion of comparative mitochondrial genomes analysis in this study. Figure S1: Maximum likelihood phylogenetic trees of the genus Tamiops constructed using various datasets. A. maximum likelihood phylogenetic tree based on the Cyt‐b dataset; B. Maximum likelihood phylogenetic tree based on the Cyt‐b + nuDNA datasets; C. maximum likelihood phylogenetic trees based on nuDNA dataset; D. maximum likelihood phylogenetic trees based on 13PCGs dataset. Figure S2: Photos of the pelage of Tamiops hainanus specimens. Figure S3: Comparative Cranial and Mandibular Characteristics of Tamiops maritimus and Tamiops hainanus. The upper section of the figure, labeled A1–A4, depicts the ventral, dorsal, and lateral views, as well as the mandibular structure of T. maritimus , respectively. The lower section, also labeled A1–A4, shows the ventral, dorsal, and lateral views, along with the mandibular structure of T. hainanus, respectively. Figure S4: Circular maps of the mitogenomes of T. swinhoei (A), T. maritimus (B), T. mcclellandii (C), and T. minshanica (D). Orange blocks represent rRNAs genes, green blocks indicate tRNAs genes, blue blocks denote PCGs, and brownish blocks illustrate the control region and origin of replication. Figure S5: Nucleotide composition of various mitogenome datasets. Hierarchical clustering of Tamiops species (y‐axis) based on nucleotide content (A) and skewness (B). Figure S6: Relative synonymous codon usage (RSCU) of mitochondrial PCGs in four species of the genus Tamiops. The proportion of each amino acid used in the construction of the 13 PCGs is displayed at the top of the bar graph. From left to right, the species represented are T. swinhoei , T. maritimus , T. mcclellandii , and T. minshanica. Figure S7: Secondary structures from the 22 tRNAs genes of the genus Tamiops. The structures of tRNAs genes are presented in the following order: (A) T. swinhoei , (B) T. maririmus, (C) T. [file ECE3-16-e73099-s001.zip › Supplement.docx]

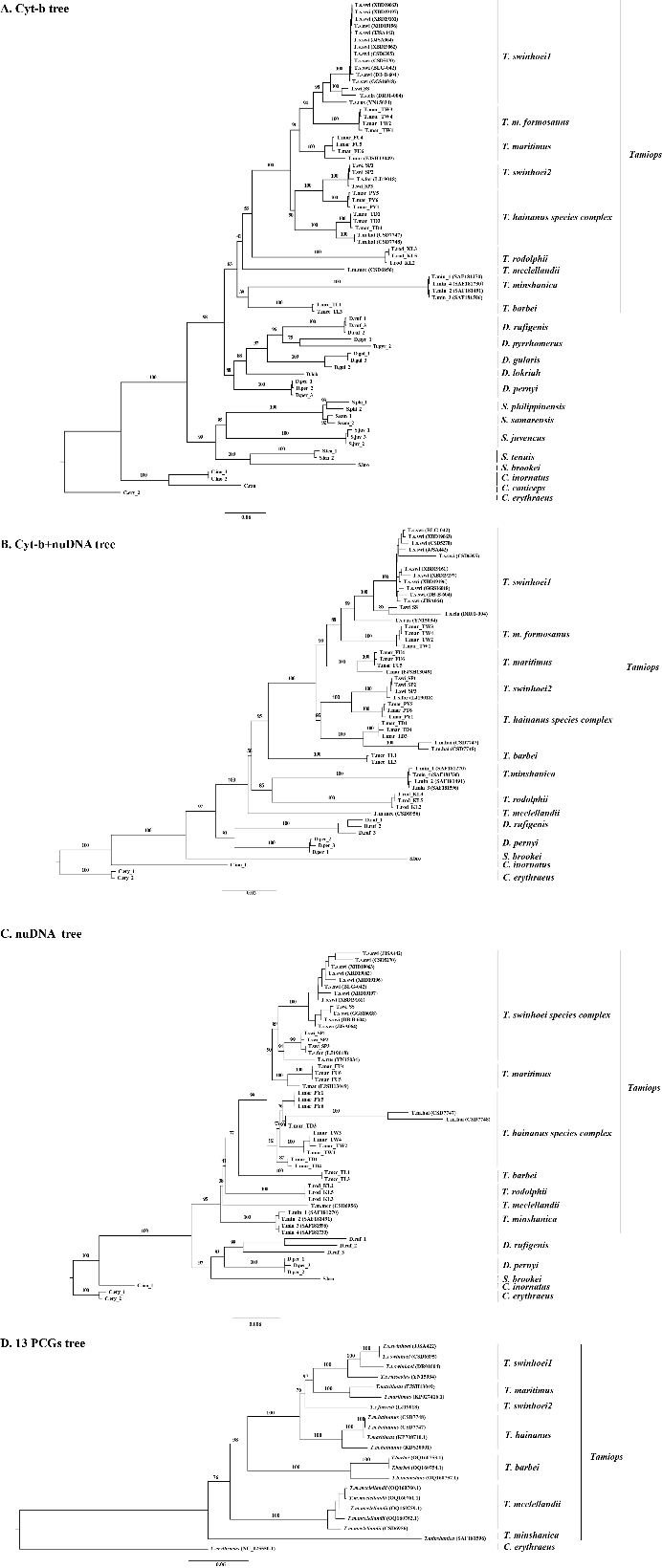


Figure S1. Maximum likelihood phylogenetic trees of the genus *Tamiops* constructed using various datasets. A. Maximum likelihood phylogenetic tree based on the Cyt-b dataset; B. Maximum likelihood phylogenetic tree based on the Cyt-b + nuDNA datasets; C. Maximum likelihood phylogenetic trees based on nuDNA dataset; D. Maximum likelihood phylogenetic trees based on 13PCGs dataset.

| Table S1. GenBank accession numbers for the DNA sequences used in the phylogenetic analyses of this study. | | | | | | | | | | |  |
| --- | --- | --- | --- | --- | --- | --- | --- | --- | --- | --- | --- |
| Museum/ GenBank No. | Species | CYTB | Sample localities | IRBP | Sample localities | RAG1 | Sample localities | PRKCI | Sample localities | Reference | |
| GenBank | *C. erythraeus* | AB499908 | Tam Dao, Vietnam | HQ698523 | Zhongxingxincun, Nantou, Taiwan | HQ698439 | Zhongxingxincun, Nantou, Taiwan | HQ698411 | Zhongxingxincun, Nantou, Taiwan | Oshida et al*.* 2001; Chang et al*.* 2011 | |
| GenBank | *C. erythraeus* | / | / | HQ698522 | Kaohsiung, Tianchi, Taiwan | HQ698438 | Kaohsiung, Tianchi, Taiwan | HQ698412 | Shishan, Nantou, Taiwan | Chang et al*.* 2011 | |
| GenBank | *C. inornatus* | AB499905 | Co Ma, Thuan, Chau, Son La, Vietnam | KX192422 | / | / | / | / | / | Oshida et al*.* 2011; Balakirev. 2016 | |
| GenBank | *C. inornatus* | AB499906 | Hon, Phu Yen, Son La, Vietnam | / | / | / | / | / | / | Oshida et al*.* 2011 | |
| GenBank | *C. caniceps* | AB499919 | Pasoh Forest Reserve, Negeri Sembilan, Malaysia | / | / | / | / | / | / | Oshida et al*.* 2011 | |
| GenBank | *Sundasciurus brookei* | KP120729 | Borneo | AY227577 | / | / | / | / | / | Tex et al. 2010; Mercer et al. 2003 | |
| GenBank | *S. juvencus* | KP120737 | Palawan, Philippines | / | / | / | / | / | / | den Tex et al. 2010 | |
| GenBank | *S. juvencus* | KP120738 | Ikadwang, Banwa Island | / | / | / | / | / | / | den Tex et al. 2010 | |
| GenBank | *S. juvencus* | KP120739 | Ikadwang, Banwa Island | / | / | / | / | / | / | den Tex et al. 2010 | |
| GenBank | *S. philippinensis* | KP120745 | Mindanao, Philippines | / | / | / | / | / | / | den Tex et al. 2010 | |
| GenBank | *S. philippinensis* | KP120746 | Mindanao, Philippines | / | / | / | / | / | / | den Tex et al. 2010 | |
| GenBank | *S. samarensis* | KP120748 | / | / | / | / | / | / | / | den Tex et al. 2010 | |
| GenBank | *S. samarensis* | KP120749 | Leyte Island | / | / | / | / | / | / | den Tex et al. 2010 | |
| GenBank | *S. tenuis* | KP120751 | Malay Peninsula | / | / | / | / | / | / | den Tex et al. 2010 | |
| GenBank | *S. tenuis* | KP120752 | Malay Peninsula | / | / | / | / | / | / | den Tex et al. 2010 | |
| GenBank | *Dremomys gularis* | EF539337 | / | / | / | / | / | / | / | Li et al. 2008 | |
| GenBank | *D. gularis* | EF539338 | / | / | / | / | / | / | / | Li et al. 2008 | |
| GenBank | *D. gularis* | EF539339 | Jingdong, Yunnan, China | / | / | / | / | / | / | Li et al. 2008 | |
| GenBank | *D. lokriah* | EF539335 | Gongshan, Yunnan, China | / | / | / | / | / | / | Li et al. 2008 | |
| GenBank | *D.pernyi* | LC150596 | Hehuanshan, Nantou, Taiwan | HQ698525 | Hehuanshan, Nantou, Taiwan | HQ698413 | Hehuanshan, Nantou, Taiwan | HQ698443 | Yilan, Nan’ao, Taiwan | Oshida et al. 2017;  Chang et al. 2011; | |
| GenBank | *D.pernyi* | HQ698361 | Hehuanshan, Nantou, Taiwan | HQ698526 | Kaohsiung, Tengzhi, Taiwan | HQ698414 | Kaohsiung:Tengzhi, Taiwan | HQ698441 | Hehuanshan,  Nantou, Taiwan | Chang et al. 2011; | |
| GenBank | *D.pernyi* | HQ698362 | Kaohsiung:Tengzhi, Taiwan | HQ698527 | Yilan, Nan’ao, Taiwan | HQ698415 | Yilan, Nan’ao, Taiwan | HQ698442 | Kaohsiung:Tengzhi, Taiwan | Chang et al. 2011; | |
| GenBank | *D.pyrrhomerus* | EF539342 | Pingbian, Yunnan, China | / | / | / | / | / | / | Li et al. 2008 | |
| GenBank | *D.pyrrhomerus* | KP708705 | / | / | / | / | / | / | / | Unpublished | |
| GenBank | *D. rufigenis* | LC150597 | Tam Dao, Vietnam | KX171260 | / | KX171273 | / | / | / | Oshida et al. 2017; Balakirev. 2016 | |
| GenBank | *D. rufigenis* | LC150598 | Tam Dao, Vietnam | KX171259 | / | KX171274 | / | / | / | Oshida et al. 2017; Balakirev. 2016 | |
| GenBank | *D. rufigenis* | LC150599 | Tam Dao, Vietnam | KX171254 | / | KX171275 | / | / | / | Oshida et al. 2017; Balakirev. 2016 | |
| GenBank | *Tamiops maritimus_FU4* | HQ698367 | Wuyishan, Fujian, China | HQ698528 | Wuyishan, Fujian, China | HQ698444 | Wuyishan, Fujian, China | HQ698416 | Wuyishan, Fujian, China | Chang et al. 2011 | |
| GenBank | *T. maritimus_FU5* | HQ698368 | Wuyishan, Fujian, China | HQ698529 | Wuyishan, Fujian, China | HQ698445 | Wuyishan, Fujian, China | HQ698417 | Wuyishan, Fujian, China | Chang et al. 2011 | |
| GenBank | *T. maritimus_FU6* | HQ698369 | Wuyishan, Fujian, China | HQ698530 | Wuyishan, Fujian, China | HQ698446 | Wuyishan, Fujian, China | HQ698418 | Wuyishan, Fujian, China | Chang et al. 2011 | |
| GenBank | *T. maritimus_TW1* | HQ698370 | Tianchi, Kaohsiung, Taiwan, China | HQ698531 | Tianchi, Kaohsiung, Taiwan, China | HQ698447 | Tianchi, Kaohsiung,Taiwan, China | HQ698419 | Tianchi, kaohsiung, Taiwan, China | Chang et al. 2011 | |
| GenBank | *T. maritimus_TW2* | HQ698371 | Taguanshan, kaohsiung, Taiwan, China | HQ698532 | Taguanshan, Kaohsiung,Taiwan,China | HQ698448 | Taguanshan,kaohsiung,Taiwan, China | HQ698420 | Taguanshan,kaohsiung,Taiwan,China | Chang et al. 2011 | |
| GenBank | *T. maritimus_TW3* | HQ698372 | Tatajia, Nantou, Taiwan, China | HQ698533 | Tatajia, Nantou, Taiwan, China | HQ698449 | Tatajia, Nantou, Taiwan, China | HQ698421 | Tatajia, Nantou, Taiwan, China | Chang et al. 2011 | |
| GenBank | *T. maritimus_TW4* | HQ698373 | Tatajia, Nantou, Taiwan, China | HQ698534 | Tatajia, Nantou, Taiwan, China | HQ698450 | Tatajia, Nantou, Taiwan, China | HQ698422 | Tatajia, Nantou, Taiwan, China | Chang et al. 2011 | |
| GenBank | *T. maritimus_TD1* | HQ698378 | Tam Dao, Vinh Phuc, Vietnam | HQ698535 | Tam Dao, Vinh Phuc, Vietnam | HQ698451 | Tam Dao, Vinh Phuc, Vietnam | HQ698423 | Tam Dao, Vinh Phuc, Vietnam | Chang et al. 2011 | |
| GenBank | *T. maritimus_TD3* | HQ698380 | Tam Dao, Vinh Phuc, Vietnam | HQ698536 | Tam Dao, Vinh Phuc, Vietnam | HQ698452 | Tam Dao, Vinh Phuc, Vietnam | HQ698424 | Tam Dao, Vinh Phuc, Vietnam | Chang et al. 2011 | |
| GenBank | *T. maritimus_TD4* | HQ698381 | Tam Dao, Vinh Phuc, Vietnam | HQ698537 | Tam Dao, Vinh Phuc, Vietnam | HQ698453 | Tam Dao, Vinh Phuc, Vietnam | HQ698425 | Tam Dao, Vinh Phuc, Vietnam | Chang et al. 2011 | |
| GenBank | *T. maritimus_PY1* | HQ698382 | Phu Yen, Sonla, Vietnam | HQ698538 | Phu Yen, Sonla, Vietnam | HQ698454 | Phu Yen, Sonla, Vietnam | HQ698426 | Phu Yen, Sonla, Vietnam | Chang et al. 2011 | |
| GenBank | *T. maritimus_PY5* | HQ698386 | Phu Yen, Sonla, Vietnam | HQ698539 | Phu Yen, Sonla, Vietnam | HQ698455 | Phu Yen, Sonla, Vietnam | HQ698427 | Phu Yen, Sonla, Vietnam | Chang et al. 2011 | |
| GenBank | *T. maritimus_PY6* | HQ698387 | Phu Yen, Sonla, Vietnam | HQ698540 | Phu Yen, Sonla, Vietnam | HQ698456 | Phu Yen, Sonla, Vietnam | HQ698428 | Phu Yen, Sonla, Vietnam | Chang et al. 2011 | |
| GenBank | *T. mcclellandii_TL1* | HQ698388 | Cheng Rai, Thailand | HQ698541 | Cheng Rai, Thailand | HQ698457 | Cheng Rai, Thailand | HQ698429 | Cheng Rai, Thailand | Chang et al. 2011 | |
| GenBank | *T. mcclellandii_TL3* | HQ698390 | Mae Chan, Thailand | HQ698542 | Mae Chan, Thailand | HQ698458 | Mae Chan, Thailand | HQ698430 | Mae Chan, Thailand | Chang et al. 2011 | |
| GenBank | *T. rodolphii_KL2* | HQ698395 | kien Luong, Kien Giang, Vietnam | HQ698543 | kien Luong, kien Giang, Vietnam | HQ698459 | kien Luong, kien Giang, Vietnam | HQ698431 | kien Luong, kien Giang, Vietnam | Chang et al. 2011 | |
| GenBank | *T. rodolphii_KL3* | HQ698396 | kien Luong, Kien Giang, Vietnam | HQ698544 | kien Luong, kien Giang, Vietnam | HQ698460 | kien Luong, kien Giang, Vietnam | HQ698432 | kien Luong, kien Giang, Vietnam | Chang et al. 2011 | |
| GenBank | *T. rodolphii_KL5* | HQ698398 | kien Luong, Kien Giang, Vietnam | HQ698545 | kien Luong, kien Giang, Vietnam | HQ698461 | kien Luong, kien Giang, Vietnam | HQ698433 | kien Luong, kien Giang, Vietnam | Chang et al. 2011 | |
| GenBank | *T. swinhoei_SP1* | HQ698401 | Sapa, Lao Cai, Vietnam | HQ698546 | Sapa, Lao Cai, Vietnam | HQ698462 | Sapa, Lao Cai, Vietnam | HQ698434 | Sapa, Lao Cai, Vietnam | Chang et al. 2011 | |
| GenBank | *T. swinhoei_SP2* | HQ698402 | Sapa, Lao Cai, Vietnam | HQ698547 | Sapa, Lao Cai, Vietnam | HQ698463 | Sapa, Lao Cai, Vietnam | HQ698435 | Sapa, Lao Cai, Vietnam | Chang et al. 2011 | |
| GenBank | *T. swinhoei_SP3* | HQ698403 | Sapa, Lao Cai, Vietnam | HQ698548 | Sapa, Lao Cai, Vietnam | HQ698464 | Sapa, Lao Cai, Vietnam | HQ698436 | Sapa, Lao Cai, Vietnam | Chang et al. 2011 | |
| GenBank | *T. swinhoei_SS* | HQ698409 | Shimian, Sichuan, China | HQ698549 | Shimian, Sichuan, China | HQ698465 | Shimian, Sichuan, China | HQ698437 | Shimian, Sichuan, China | Chang et al. 2011 | |
| GenBank | *Tamiops minshanica* | OK432502 | Wanglang, Sichuan, China | OK432506 | Wanglang, Sichuan, China | OK432509 | Wanglang, Sichuan, China | OK432512 | Wanglang, Sichuan, China | Liu et al. 2022 | |
| GenBank | *T. minshanica* | OK432501 | Wanglang, Sichuan, China | OK432505 | Wanglang, Sichuan, China | OK432508 | Wanglang, Sichuan, China | OK432511 | Wanglang, Sichuan, China | Liu et al. 2022 | |
| GenBank | *T. minshanica* | OK432499 | Wanglang, Sichuan, China | OK432503 | Wanglang, Sichuan, China | OK432507 | Wanglang, Sichuan, China | OK432510 | Wanglang, Sichuan, China | Liu et al. 2022,  In this study | |
| GenBank | *T. s. swinhoei* | OK493503 | Baoxing, Sichuan, China | OK493505 | Baoxing, Sichuan, China | OK493507 | Baoxing, Sichuan, China | OK493509 | Baoxing, Sichuan, China | Liu et al*.* 2022 | |
|  |  |  |  |  |  |  |  |  |  |  | |
| SAF201148 | *Tamiops maritimus hainanus* | PQ591913 | Hainan, China | / | Hainan, China | PQ629581 | Hainan, China | PQ629564 | Hainan, China | In this study | |
| CSD7748 | *T. m. hainanus* | PQ591914 | Hainan, China | PQ629549 | Hainan, China | PQ629582 | Hainan, China | PQ629565 | Hainan, China | In this study | |
| CSD6956 | *Tamiops mcclellandii mcclellandii* | PQ591912 | Motuo, Xizang, China | PQ629548 | Motuo, Xizang, China | PQ629580 | Motuo, Xizang, China | PQ629563 | Motuo, Xizang, China | In this study | |
| SAF181596 | *Tamiops minshanica* | OK432500 | Wanglang, Sichuan, China | OK432504 | Wanglang, Sichuan, China | / | / | / | / | Liu et al. 2022,  In this study | |
| SAF19631 | *T. s. swinhoei* | PQ629532 | Pingwu, Sichuan, China | PQ629541 | Pingwu, Sichuan, China | PQ629573 | Pingwu, Sichuan, China | PQ629556 | Pingwu, Sichuan, China | In this study | |
| SAF19632 | *T. s. swinhoei* | PQ629533 | Pingwu, Sichuan, China | PQ629542 | Pingwu, Sichuan, China | PQ629574 | Pingwu, Sichuan, China | PQ629557 | Pingwu, Sichuan, China | In this study | |
| SAF19730 | *T. s. swinhoei* | PQ629534 | Pingwu, Sichuan, China | PQ629543 | Pingwu, Sichuan, China | PQ629575 | Pingwu, Sichuan, China | PQ629558 | Pingwu, Sichuan, China | In this study | |
| SAF19765 | *T. s. swinhoei* | PQ629535 | Pingwu, Sichuan, China | PQ629544 | Pingwu, Sichuan, China | PQ629576 | Pingwu, Sichuan, China | PQ629559 | Pingwu, Sichuan, China | In this study | |
| SAF19766 | *T. s. swinhoei* | PQ629536 | Pingwu, Sichuan, China | PQ629545 | Pingwu, Sichuan, China | PQ629577 | Pingwu, Sichuan, China | PQ629560 | Pingwu, Sichuan, China | In this study | |
| SAF09282 | *T. s. swinhoei* | OK493504 | Baoxing, Sichuan, China | OK493506 | Baoxing, Sichuan, China | OK493508 | Baoxing, Sichuan, China | OK493510 | Baoxing, Sichuan, China | Liu et al. 2022,  In this study | |
| CSD5270 | *T. s. swinhoei* | PQ629537 | Emeishan, Sichuan, China | PQ629546 | Emeishan, Sichuan, China | PQ629578 | Emeishan, Sichuan, China | PQ629561 | Emeishan, Sichuan, China | In this study | |
| CSD6395 | *T. s. swinhoei* | PQ591911 | Emeishan, Sichuan, China | PQ629547 | Emeishan, Sichuan, China | PQ629579 | Emeishan, Sichuan, China | PQ629562 | Emeishan, Sichuan, China | In this study | |
| SAF11622 | *T. s. swinhoei* | PQ629529 | Xiaojin, Sichuan, China | PQ629538 | Xiaojin, Sichuan, China | PQ629570 | Xiaojin, Sichuan, China | PQ629553 | Xiaojin, Sichuan, China | In this study | |
| SAF16601 | *T. s. swinhoei* | PQ629530 | Minya Konka, Sichuan, China | PQ629539 | Minya Konka, Sichuan, China | PQ629571 | Minya Konka, Sichuan, China | PQ629554 | Minya Konka, Sichuan, China | In this study | |
| SAF05486 | *T. s. swinhoei* | PQ629531 | Danba, Sichuan, China | PQ629540 | Danba, Sichuan, China | PQ629572 | Danba, Sichuan, China | PQ629555 | Danba, Sichuan, China | In this study | |
| SAF14745 | *T. s. clarkei* | PQ591915 | Derong, Sichuan, China | / | Derong, Sichuan, China | PQ629583 | Derong, Sichuan, China | PQ629566 | Derong, Sichuan, China | In this study | |
| SAF15254 | *T. s. russeolus* | PQ591920 | Deqin, Yunnan, China | PQ629552 | Deqin, Yunnan, China | PQ629586 | Deqin, Yunnan, China | PQ629569 | Deqin, Yunnan, China | In this study | |
| SAF19814 | *T. s. forresti* | PQ591918 | Lijiang, Yunnan, China | PQ629551 | Lijiang, Yunnan, China | PQ629585 | Lijiang, Yunnan, China | PQ629568 | Lijiang, Yunnan, China | In this study | |
| SAF13049 | *T. maritimus* | PQ591916 | Tongren, Guizhou, China | PQ629550 | Tongren, Guizhou, China | PQ629584 | Tongren, Guizhou, China | PQ629567 | Tongren, Guizhou, China | In this study | |

Reference:

Balakirev AE (2016) Phylogeography and taxonomy of Asian red-cheeked squirrels (Rodentia, Sciuridae, *Dremomys*) in Vietnam. Zootaxa.

Chang SW, Oshida T, Endo H, Nguyen ST, Dang CN, Nguyen DX, Jiang, XL, Li ZJ, Lin LK (2011) Ancient hybridization and underestimated species diversity in Asian striped squirrels (genus *Tamiops*): inference from paternal, maternal and biparental markers. Journal of Zoology 285: 128–138.

Tex RJ, Thorington R, Maldonado JE, Leonard JA (2010) Speciation dynamics in the SE Asian tropics: Putting a time perspective on the phylogeny and biogeography of Sundaland tree squirrels, *Sundasciurus*. Molecular phylogenetics and evolution 55(2): 711–720.

Song L, Yu F, Su Y, Wang Y, Jiang X, Mcguire PM, Feng Q, Yang JX (2008) Molecular phylogeny of five species of *Dremomys* (Rodentia: Sciuridae), inferred from cytochrome b gene sequences. Zoologica Scripta 37(4): 349-354.

Mercer JM, Roth VL (2003) The effects of Cenozoic global change on squirrel phylogeny. Science 299: 1568-1572.

Oshida T, Dang CN, Nguyen ST, Nguyen NX, Hayashi Y (2011) Phylogenetic relationship between *Callosciurus caniceps* and *C. inornatus* (Rodentia, Sciuridae): implications for zoogeographical isolation by the Mekong River. Italian Journal of Zoology 78: 328-335.

Oshida T, Lin LK, Chang SW, Dang CN, Nguyen ST, Nguyen NX, Nguyen DX, Endo H, Kimura J, Sasaki M (2017) Mitochondrial DNA evidence reveals genetic difference between Perny's long-nosed squirrels in Taiwan and Asian mainland. Mammal Study 42:111-116.

| Table S2. Sequence lengths of each gene and the best substitution models employed to reconstruct the phylogenetic tree. | | |
| --- | --- | --- |
| Partitions | Sequence length | best model |
| Cyt-b | 1140 | GTR+I+G |
| IRBP | 1016 | GTR+I+G |
| RAG1 | 1190 | GTR+I+G |
| PRKCI | 436 | GTR+I+G |
|  |  |  |
| Table S3. Sequence lengths of 13 PCGs and the best substitution model employed for reconstructing the phylogenetic tree. | | |
| Partitions | Sequence length | best model |
| ND1 | 957 | HKY+I+G |
| ND2 | 1044 | GTR+I+G |
| COX1 | 1542 | GTR+I+G |
| COX2 | 684 | HKY+I |
| ATP8 | 204 | HKY+G |
| ATP6 | 681 | HKY+I+G |
| COX3 | 785 | HKY+I+G |
| ND3 | 348 | HKY+G |
| ND4L | 297 | HKY+G |
| ND4 | 1378 | HKY+I+G |
| ND5 | 1818 | GTR+G |
| ND6 | 525 | GTR+I+G |
| Cyt-b | 1140 | GTR+I+G |

| Table S4. External and skull measurements of new additions to species within the genus *Tamiops* | | | | | | | | | | | |
| --- | --- | --- | --- | --- | --- | --- | --- | --- | --- | --- | --- |
| Species | Voucher Number | HBL | TL | HFL | GLS | PL | ZOB | IOB | LUD | LMXTR |  |
| *T.swinhoei* | CSD6395 | 141 | 120 | 32 | 39.46 | 15.14 | 22.46 | 13.12 | 8.74 | 6.78 |  |
| *T.swinhoei* | CSD5270 | 152 | 115 | 36 | 39.08 | 15.85 | 22.44 | / | 8.84 | 6.7 |  |
| *T.swinhoei* | 820274 | 145 | 120 | 33 | 38.55 | 16.21 | 23.18 | 13.73 | 8.92 | 6.8 |  |
| *T.swinhoei* | 17267 | 134 | 117 | 35 | 39.51 | 15.48 | 22.72 | 12.97 | 9.06 | 6.71 |  |
| *T.swinhoei* | 17264-1 | 149 | 101 | 31 | 38.53 | 15.31 | 22.98 | 12.89 | 8.49 | 6.78 |  |
| *T.swinhoei* | 28081 | 135 | 130 | 35 | 39.04 | 16.24 | 22.37 | 12.6 | 9.94 | 6.69 |  |
| *T.swinhoei* | 17264 | 149 | 101 | 31 | 39.77 | 16.65 | 23.29 | 13.06 | 9.27 | 6.78 |  |
| *T.swinhoei* | 17262 | 140 | 110 | 30 | 40.86 | 16.89 | 22.55 | 12.49 | 9.85 | 6.75 |  |
| *T.swinhoei* | 28082 | 148 | 120 | 35 | 40.54 | 16.47 | 22.36 | 13.26 | 9.89 | 6.7 |  |
| *T.swinhoei* | JJSA422 | 150 | 105 | 34 | 39.37 | 15.81 | 23.74 | 13.33 | 8.82 | 6.89 |  |
| *T.swinhoei* | XBD19062 | 145 | 107 | 36 | 40.44 | 16.67 | 23.65 | 13.81 | 8.72 | 7.65 |  |
| *T.swinhoei* | XBD19063 | 150 | 120 | 35 | 41.5 | 16.57 | 23.11 | 13.4 | 9.18 | 7.42 |  |
| *T.swinhoei* | XBD19196 | 152 | 113 | 36 | 40.01 | 16.27 | 23.47 | 12.83 | 8.71 | 7.32 |  |
| *T. maritimus* | 3007 | 130 | 105 | 27 | 36.74 | 15.37 | 22.37 | 12.57 | 9.08 | 6.39 |  |
| *T. maritimus* | 3040 | 135 | 110 | 20 | 37.24 | 15.58 | 22.05 | 12.59 | 8.35 | 6.51 |  |
| *T. maritimus* | 2185 | 140 | 95 | 25 | 37.03 | 15.5 | 22.36 | 12.68 | 9.03 | 6.33 |  |
| *T. maritimus* | 783 | 119 | 112 | 28 | 35.73 | 15 | 20.53 | 12.02 | 8.26 | 6.41 |  |
| *T. maritimus* | 769 | 136 | 100 | 21 | 37.23 | 15.54 | 21.91 | 12.02 | 8.5 | 6.47 |  |
| *T. maritimus* | 885 | 135 | 128 | 28 | 36.43 | 15.08 | 21.59 | 13.43 | 8.62 | 6.3 |  |
| *T. maritimus* | 782 | 131 | 111 | 29 | 35.6 | 15.07 | 21.57 | 12.8 | 9.15 | 5.9 |  |
| *T. maritimus* | 839 | 117 | 113 | 31 | 36.38 | 15.14 | 22.11 | 13.19 | 8.66 | 6.48 |  |
| *T. maritimus* | 838 | 117 | 121 | 29 | 35.61 | 15.08 | 21.56 | 13.12 | 8.18 | 6.39 |  |
| *T. maritimus* | 864 | 126 | 125 | 29 | 36.52 | 15.18 | 22.21 | 12.65 | 8.76 | 6.26 |  |
| *T. maritimus* | 756 | 135 | 112 | 32 | 36.45 | 14.78 | 21.28 | 12.69 | 8.23 | 6.35 |  |
| *T. maritimus* | 767 | 135 | 105 | 21 | 36.37 | 15.23 | 22.43 | 13.37 | 8.52 | 6.61 |  |
| *T. maritimus* | 117 | 127 | 106 | 29 | 37.3 | 15.01 | 22.3 | 13.38 | 8.31 | 6.57 |  |
| *T. maritimus* | 505 | 132 | 100 | 31 | 36.76 | 14.74 | 21.38 | 12.82 | 8.42 | 6.12 |  |
| *T. maritimus* | 46 | 128 | 101 | 30 | 35.08 | 15.07 | 21.03 | 12.38 | 8.29 | 6.47 |  |
| *T. maritimus* | 922 | 136 | 104 | 31 | 36.9 | 15.7 | 21.71 | 13.3 | 9.23 | 6.31 |  |
| *T. maritimus* | 919 | 127 | 115 | 28 | 35.64 | 14.85 | 21.21 | 13.09 | 8.59 | 6.23 |  |
| *T. maritimus* | 2001 | 110 | 110 | 28 | 35.37 | 15.38 | 22.16 | 12.86 | 8.16 | 6.74 |  |
| *T. maritimus* | 30 | 129 | 93 | 31 | 37.04 | 15.5 | 22.83 | 12.71 | 8.61 | 6.38 |  |
| *T. maritimus* | 88 | 132 | 97 | 26 | 36.55 | 15.06 | 21.67 | 12.35 | 8.42 | 6.26 |  |
| *T. maritimus* | 866 | 125 | 116 | 26 | 35.72 | 15.01 | 21 | 12.37 | 8.23 | 6.59 |  |
| *T. maritimus* | 79 | 131 | 102 | 29 | 36.6 | 15.23 | 22.05 | 13.33 | 8.48 | 6.36 |  |
| *T. maritimus* | 802 | 138 | 113 | 28 | 37.08 | 14.95 | 22.25 | 12.4 | 8.42 | 6.41 |  |
| *T. maritimus* | 903 | 122 | 89 | 27 | 37.84 | 16.27 | 22.39 | 13 | 10.18 | 6.4 |  |
| *T. maritimus* | 837 | 121 | 116 | 29 | 35.27 | 14.62 | 21.79 | 12.92 | 8.13 | 6.41 |  |
| *T. maritimus* | 902 | 133 | 122 | 29 | 37.2 | 15.67 | 22.1 | 13.31 | 9.06 | 6.29 |  |
| *T. maritimus* | 875 | 135 | 117 | 28 | 37.33 | 15.55 | 22.1 | 12.91 | 8.48 | 6.72 |  |
| *T. maritimus* | 827 | 131 | 97 | 30 | 35.55 | 14.71 | 22.33 | 12.98 | 8.27 | 6.47 |  |
| *T. maritimus* | o683 | 104 | 107 | 26 | 33.44 | 13.26 | 19.95 | 11.68 | 7.25 | 6.1 |  |
| *T. maritimus* | 477 | 120 | 104 | 27 | 35.37 | 14.03 | 19.81 | 12.05 | 7.84 | 6.14 |  |
| *T. maritimus* | 22749 | 100 | 99 | 30 | 33.91 | 14.26 | 19.43 | 12.03 | 8.24 | 5.96 |  |
| *T. maritimus* | 22808 | 138 | 105 | 30 | 36.79 | 15.01 | 22.21 | 12.96 | 8.65 | 6.2 |  |
| *T. maritimus* | 22804 | 105 | 105 | 30 | 36.35 | 14.92 | 21.23 | 12.17 | 8.75 | 6.18 |  |
| *T. maritimus* | 22796 | 120 | 95 | 28 | 36.49 | 14.71 | 21.83 | 12.62 | 8.45 | 6.02 |  |
| *T. maritimus* | 22803 | 115 | 100 | 31 | 36.78 | 15.74 | 21.93 | 12.76 | 9.29 | 6.29 |  |
| *T. hainanus* | o017 | 114 | 107 | 22 | 33.2 | 13.38 | 19.97 | 11.57 | 7.56 | 6.31 |  |
| *T. hainanus* | o156 | 120 | 107 | 26 | 34.76 | 13.12 | 20.34 | 12.59 | 7.33 | 6.33 |  |
| *T. hainanus* | o412 | 119 | 106 | 26 | 33.21 | 13.65 | 20.91 | 12.16 | 7.53 | 6.03 |  |
| *T. hainanus* | o095 | 106 | 110 | 25 | 33.14 | 13.79 | 19.56 | 11.49 | 7.11 | 6.15 |  |
| *T. hainanus* | o259 | 125 | 120 | 26 | 34.58 | 14.33 | 20.79 | 12.74 | 8.18 | 6.02 |  |
| *T. hainanus* | o159 | 120 | 98 | 28 | 33.64 | 13.86 | 20.95 | 12.36 | 7.69 | 6.24 |  |
| *T. hainanus* | o096 | 104 | 101 | 24 | 33.61 | 13.98 | 19.82 | 11.56 | 7.38 | 6.35 |  |
| *T. hainanus* | 12923 | 118 | 101 | 26 | 33.09 | 13.44 | 18.96 | 11.27 | 7.42 | 5.97 |  |
| *T. hainanus* | 12922 | 123 | 107 | 28 | 34.6 | 14.99 | 21.03 | 12.81 | 8.37 | 6.3 |  |
| *T. hainanus* | 12924 | 125 | 103 | 29 | 33.54 | 13.73 | 20.46 | 13.13 | 7.73 | 6.12 |  |
| *T. hainanus* | 12920 | 116 | 98 | 28 | 33.66 | 14.8 | 19.89 | 11.9 | 8.22 | 6.1 |  |
| *T. hainanus* | 12977 | 123 | 98 | 29 | 34.68 | 14.32 | 21.36 | 12.57 | 7.99 | 6.16 |  |
| *T. hainanus* | 12967 | 112 | 106 | 26 | 32.7 | 13.29 | 19.6 | 10.99 | 7.34 | 6.02 |  |
| *T. hainanus* | 12987 | 113 | 105 | 26 | 33.38 | 13.5 | 21.25 | 11.69 | 7.76 | 5.51 |  |
| *T. hainanus* | 12976 | 124 | 109 | 26 | 34.85 | 14.69 | 20.47 | 12.11 | 8.37 | 6.08 |  |
| *T. hainanus* | 12990 | 111 | 103 | 26 | 32.63 | 13.38 | 19.79 | 11.74 | 7.38 | 5.98 |  |
| *T. hainanus* | 12993 | 117 | 109 | 27 | 33.43 | 13.39 | 19.97 | 12.29 | 7.57 | 5.6 |  |
| *T.rodophii* | 1903 | 118 | 102 | 29 | 32.6 | 13 | 21 | 12.5 | 7 | 5.5 |  |
| *T.rodophii* | 1904 | 115 | 120 | 29.5 | 32 | 12.2 | 20.1 | 11.9 | 6.8 | 5.5 |  |
| *T.rodophii* | 2337 | 118 | 117 | 27.5 | 32 | 12 | 19.5 | 12 | 6.2 | 6.1 |  |
| *T.rodophii* | 2040 | 138 | 119 | 29 | 32.6 | 13.2 | 20.8 | 11.8 | 7.6 | 5.9 |  |
| *T.rodophii* | 294876 | 120 | 130 | 30 | 32.6 | 15.8 | 20.1 | 13.6 | 8.6 | 5.4 |  |
| *T. barbei* | 14864 | 112 | 114 | 26 | 32.57 | 12.99 | 19.48 | 11.94 | 7.2 | 5.63 |  |
| *T. barbei* | 14844 | 112 | 113 | 24 | 32.82 | 13.01 | 19.31 | 11.38 | 7.1 | 5.77 |  |
| *T. barbei* | 14843 | 113 | 104 | 25 | 32.59 | 13.01 | 19.4 | 11.66 | 7.31 | 5.47 |  |
| *T. barbei* | 14852 | 105 | 100 | 25 | 32.1 | 13.01 | 18.95 | 11.49 | 6.92 | 5.43 |  |
| *T. barbei* | 14846 | 108 | 99 | 26 | 31.43 | 12.71 | 19.17 | 11.76 | 6.68 | 5.78 |  |
| *T. barbei* | 14827 | 108 | 100 | 23 | 31.9 | 12.91 | 18.73 | 11.3 | 6.75 | 5.53 |  |
| *T. barbei* | 14878 | 110 | 110 | 20 | 32.64 | 12.78 | 19.38 | 11.61 | 7.14 | 5.62 |  |
| *T. barbei* | 14872 | 110 | 105 | 27 | 32.27 | 12.96 | 20.03 | 12.42 | 7.55 | 5.49 |  |
| *T. barbei* | 14870 | 109 | 98 | 22 | 31.93 | 13.42 | 19.82 | 11.83 | 7.34 | 5.42 |  |
| *T. barbei* | 14868 | 112 | 105 | 27 | 32.48 | 13.22 | 20.09 | 11.55 | 7.64 | 5.67 |  |
| *T. barbei* | 76288 | 100 | 96 | 23 | 29.78 | 12.99 | 17.23 | 10.12 | 7.44 | 5.34 |  |
| *T. barbei* | 76301 | 100 | 95 | 22 | 31.3 | 13.19 | 18.61 | 11.05 | 7.61 | 5.29 |  |
| *T. barbei* | 76296 | 110 | 95 | 22 | 32.41 | 13.52 | 18.67 | 10.98 | 8.07 | 5.21 |  |
| *T. barbei* | 76295 | 113 | 96 | 23 | 31.93 | 13.34 | 19.38 | 11.92 | 7.82 | 5.74 |  |
| *T. barbei* | 76258 | 105 | 100 | 24 | 32.39 | 13.39 | 19.46 | 11.39 | 7.84 | 5.34 |  |
| *T. barbei* | 640210 | 105 | 106 | 25 | 31.11 | 12.99 | 18.03 | 11.17 | 6.98 | 5.53 |  |
| *T. barbei* | 516 | 110 | 106 | 27 | 32.12 | 13.15 | 19.13 | 11.31 | 7.27 | 5.61 |  |
| *T. barbei* | 620039 | 103 | 94 | 24 | 32.44 | 12.97 | 19.49 | 11.14 | 7.27 | 5.53 |  |
| *T. barbei* | 620056 | 115 | 100 | 25 | 31.89 | 13.05 | 19.34 | 11.73 | 7.37 | 5.16 |  |
| *T. barbei* | 80083 | 107 | 104 | 22 | 32.44 | 13.42 | 19.49 | 11.54 | 7.42 | 5.41 |  |
| *T. barbei* | 776 | 110 | 107 | 27 | 32.15 | 12.99 | 18.72 | 12.24 | 7.25 | 5.6 |  |
| *T. barbei* | Z000551 | 106 | 105 | 25 | 33.33 | 13.35 | 19.8 | 11.99 | 7.84 | 5.32 |  |
| *T. barbei* | 302 | 117 | 101 | 25 | 33.02 | 13.48 | 19.72 | 11.99 | 7.69 | 5.15 |  |

| Table S5 Factor loadings and percentage of variance explained for principal component analysis on *Tamiops* species. | | |
| --- | --- | --- |
| Variables | Factor component | |
|  | PC1 | PC2 |
| GLS | 0.969 | -0.07 |
| ZOB | 0.937 | -0.056 |
| HBL | 0.874 | 0.049 |
| LUD | 0.857 | -0.245 |
| IOB | 0.851 | 0.02 |
| PL | 0.766 | -0.363 |
| LMXTR | 0.761 | 0.116 |
| HFL | 0.621 | 0.494 |
| TL | 0.178 | 0.861 |
| eigenvalues | 5.624 | 1.203 |
| Variance explained (%) | 62.486 | 13.361 |

| Table S6. General features of the mitochondrial genome of the four species within the genus *Tamiops,* left to right: *T. swinhoei*, *T. maritimus*, *T. mcclellandii*, and *T. minshanica*, respectively. | | | | | | | | |
| --- | --- | --- | --- | --- | --- | --- | --- | --- |
| Gene | Position | | Orientation | Size (bp) | Intergenic Nucleotides | Anticodon | Codon | |
|  | Start | Stop |  |  |  |  | Start codon | Stop codon |
| tRNA^phe^ | 1/1/1/1 | 68/68/72/72 | +/+/+/+ | 68/68/72/72 | 0/0/0/0 | gaa/gaa/gaa/gaa |  |  |
| rrnS | 69/69/73/73 | 1031/1032/1038/1039 | +/+/+/+ | 963/964/966/967 | 0/0/0/0 |  |  |  |
| tRNA^Val^ | 1032/1033/1039/1040 | 1103/1105/1109/1110 | +/+/+/+ | 72/73/71/71 | 0/0/0/0 | tac/tac/tac/tac |  |  |
| rrnL | 1104/1106/1110/1111 | 2679/2681/2686/2686 | +/+/+/+ | 1576/1576/1577/1576 | 0/0/0/0 |  |  |  |
| tRNA^Leu^2 | 2680/2682/2687/2687 | 2754/2756/2761/2761 | +/+/+/+ | 75/75/75/75 | 3/3/3/3 | taa/taa/taa/taa |  |  |
| ND1 | 2758/2760/2765/2765 | 3714/3716/3721/3721 | +/+/+/+ | 957/957/957/957 | -1/-1/0/0 |  | ATG/ATG/ATG/ATG | TAA/TAA/TAA/TAA |
| tRNA^Ile^ | 3714/3716/3722/3722 | 3782/3784/3790/3790 | +/+/+/+ | 69/69/69/69 | -3/-3/-3/-3 | gat/gat/gat/gat |  |  |
| tRNA^Gln^ | 3780/3782/3788/3788 | 3851/3853/3859/3859 | -/-/-/- | 72/72/72/72 | 2/2/2/2 | ttg/ttg/ttg/ttg |  |  |
| tRNA^Met^ | 3854/3856/3862/3862 | 3924/3926/3930/3931 | +/+/+/+ | 71/71/69/70 | 0/0/0/0 | cat/cat/cat/cat |  |  |
| ND2 | 3925/3927/3931/3932 | 4968/4970/4974/4975 | +/+/+/+ | 1044/1044/1044/1044 | -2/-2/-2/-2 |  | ATT/ATT/ATT/ATC | TAG/TAG/TAG/TAG |
| tRNA^Trp^ | 4967/4969/4973/4974 | 5033/5035/5039/5039 | +/+/+/+ | 67/67/67/66 | 3/3/3/4 | tca/tca/tca/tca |  |  |
| tRNA^Ala^ | 5037/5039/5043/5044 | 5105/5107/5111/5112 | -/-/-/- | 69/69/69/69 | 6/6/5/7 | tgc/tgc/tgc/tgc |  |  |
| tRNA^Asn^ | 5112/5114/5117/5120 | 5184/5186/5189/5192 | -/-/-/- | 73/73/73/73 | 2/2/2/2 | gtt/gtt/gtt/gtt |  |  |
| OL | 5187/5189/5192/5195 | 5215/5218/5221/5223 | +/+/+/+ | 29/30/30/29 | -1/-1/-1/-1 |  |  |  |
| tRNA^Cys^ | 5215/5218/5221/5223 | 5281/5284/5287/5289 | -/-/-/- | 67/67/67/67 | 1/1/1/0 | gca/gca/gca/gca |  |  |
| tRNA^Tyr^ | 5283/5286/5289/5290 | 5348/5351/5354/5355 | -/-/-/- | 66/66/66/66 | 8/8/8/8 | gta/gta/gta/gta |  |  |
| COX1 | 5357/5360/5363/5364 | 6898/6901/6904/6905 | +/+/+/+ | 1542/1542/1542/1542 | 2/2/2/2 |  | ATG/ATG/ATG/ATG | TAG/TAA/TAG/TAA |
| tRNA^Ser^2 | 6901/6904/6907/6908 | 6969/6972/6975/6976 | -/-/-/- | 69/69/69/69 | 3/3/3/3 | tga/tga/tga/tga |  |  |
| tRNA^Asp^ | 6973/6976/6979/6980 | 7041/7044/7047/7048 | +/+/+/+ | 69/69/69/69 | 1/1/1/1 | gtc/gtc/gtc/gtc |  |  |
| COX2 | 7043/7046/7049/7050 | 7726/7729/7732/7733 | +/+/+/+ | 684/684/684/684 | 3/3/3/3 |  | ATG/ATG/ATG/ATG | TAA/TAA/TAA/TAA |
| tRNA^Lys^ | 7730/7733/7736/7737 | 7797/7800/7803/7802 | +/+/+/+ | 68/68/68/66 | 1/1/1/1 | ttt/ttt/ttt/ttt |  |  |
| ATP8 | 7799/7802/7805/7804 | 8002/8005/8008/8007 | +/+/+/+ | 204/204/204/204 | -43/-43/-43/-43 |  | ATG/ATG/ATG/ATG | TAA/TAA/TAA/TAA |
| ATP6 | 7960/7963/7966/7965 | 8640/8643/8646/8645 | +/+/+/+ | 681/681/681/681 | -1/-1/-1/-1 |  | ATG/ATG/ATG/ATG | TAA/TAA/TAA/TAA |
| COX3 | 8640/8643/8646/8645 | 9424/9427/9430/9429 | +/+/+/+ | 785/785/785/785 | -1/-1/-1/-1 |  | ATG/ATG/ATG/ATG | TA/TA/TA/TA |
| tRNA^Gly^ | 9424/9427/9430/9429 | 9494/9498/9500/9499 | +/+/+/+ | 71/72/71/71 | 0/0/0/0 | tcc/tcc/tcc/tcc |  |  |
| ND3 | 9495/9499/9501/9500 | 9842/9846/9848/9847 | +/+/+/+ | 348/348/348/348 | 0/0/0/0 |  | ATA/ATA/ATA/ATT | TAA/TAA/TAA/TAA |
| tRNA^Arg^ | 9843/9847/9849/9848 | 9909/9913/9915/9914 | +/+/+/+ | 67/67/67/67 | 1/1/1/1 | tcg/tcg/tcg/tcg |  |  |
| ND4l | 9911/9915/9917/9916 | 10207/10211/10213/10212 | +/+/+/+ | 297/297/297/297 | -7/-7/-7/-7 |  | ATG/ATG/ATG/ATG | TAA/TAA/TAA/TAA |
| ND4 | 10201/10205/10207/10206 | 11578/11582/11584/11583 | +/+/+/+ | 1378/1378/1378/1378 | 0/0/0/0 |  | ATG/ATG/ATG/ATG | T/T/T/T |
| tRNA^His^ | 11579/11583/11585/11584 | 11647/11651/11653/11651 | +/+/+/+ | 69/69/69/68 | 0/0/0/0 | gtg/gtg/gtg/gtg |  |  |
| tRNA^Ser^1 | 11648/11652/11654/11652 | 11707/11711/11713/11712 | +/+/+/+ | 60/60/60/61 | 0/0/0/0 | gct/gct/gct/gct |  |  |
| tRNA^Leu^1 | 11708/11712/11714/11713 | 11777/11781/11783/11782 | +/+/+/+ | 70/70/70/70 | 0/0/0/0 | tag/tag/tag/tag |  |  |
| ND5 | 11778/11782/11784/11783 | 13595/13599/13601/13600 | +/+/+/+ | 1818/1818/1818/1818 | -17/-17/-17/-17 |  | ATA/ATA/ATA/ATA | TAA/TAA/TAA/TAA |
| ND6 | 13579/13583/13585/13584 | 14103/14107/14109/14108 | -/-/-/- | 525/525/525/525 | 0/0/0/0 |  | ATG/ATG/ATG/ATG | AGG/AGA/AGG/AGA |
| tRNA^Glu^ | 14104/14108/14110/14109 | 14172/14176/14178/14177 | -/-/-/- | 69/69/69/69 | 4/4/4/5 | ttc/ttc/ttc/ttc |  |  |
| Cyt-b | 14177/14181/14183/14183 | 15316/15320/15322/15322 | +/+/+/+ | 1140/1140/1140/1140 | 1/1/1/1 |  | ATG/ATG/ATG/ATG | AGA/AGA/AGA/AGA |
| tRNA^Thr^ | 15318/15322/15324/15324 | 15386//15390/15393/15392 | +/+/+/+ | 69/69/70/69 | 3/3/3/3 | tgt/tgt/tgt/tgt |  |  |
| tRNA^pro^ | 15390/15394/15397/15396 | 15458/15462/15465/15464 | -/-/-/- | 69/69/69/69 | 0/0/0/0 | tgg/tgg/tgg/tgg |  |  |
| D-loop | 15459/15463/15466/15465 | 16501/16523/16523/16559 | +/+/+/+ | 1043/1061/1058/1095 | 0/0/0/0 |  |  |  |

Appendix I: Results and discussion of comparative mitochondrial genomes analysis in this study

**Results**

Genome organization and composition

The eleven complete mitogenomes of four species within the genus *Tamiops* have been successfully assembled and characterized as classical circular double-stranded DNA molecules. Their total lengths range from 16,501 bp in *T. swinhoei* to 16,559 bp in *T. minshanica* (Table S6). The complete mitogenomes of *Tamiops* species consist of 37 coding regions: 13 PCGs, 22 tRNA genes, 2 rRNA genes, 1 non-coding region (the D-loop) and the origin of L-strand replication (OL) (Table S6, Fig. 4). A total of 12 PCGs and 14 tRNA genes are located on the heavy (H) strand, while the light (L) strand encodes NADH dehydrogenase subunit 6 (ND6), tRNA *^Gln^*, tRNA *^Ala^*, tRNA *^Asn^*, tRNA *^Cys^*, tRNA *^Tyr^*, tRNA *^Ser2^*, tRNA *^Glu^* and tRNA *^Pro^* (Table S6, Fig. S4). The mitogenome of the genus *Tamiops* contains 8 or 9 overlapping regions, ranging from 1 to 43 bp, and 15 or 16 intergenic spacers, ranging from 1 to 8 bp. The two largest overlapping regions are located between ATP8 and ATP6 (43 bp) and between ND5 and ND6 (17 bp). The analysis of nucleotide composition in the mitogenomes of four species within the genus *Tamiops*, when combined with published sequences, reveals a bias toward adenine (A, 32.17% – 32.71%) and thymine (T, 26.72% – 31.57%). Additionally, a higher preference for adenine- thymine (AT) content was observed (Fig. S5A), along with GC-skew and AT-skew regions (Fig. S5B) across all known mitogenome sequences. The G + C content and A + T content of the mitogenomes were calculated to range from 37.9% to 40.75% and 59.25% to 62.01%, respectively (Fig. S5A). Notably, the G + C content is lower than the A + T content. Furthermore, a more pronounced nucleotide bias was detected in the complete mitogenomes, and, similar to most known vertebrates, the GC-skew is negative.

Figure S4. Circular maps of the mitogenomes of *T. swinhoei* (A), *T. maritimus* (B), *T. mcclellandii* (C), and *T. minshanica* (D). Orange blocks represent rRNAs genes, green blocks indicate tRNAs genes, blue blocks denote PCGs, and brownish blocks illustrate the control region and origin of replication.

Figure S5. Nucleotide composition of various mitogenome datasets. Hierarchical clustering of *Tamiops* species (y-axis) based on nucleotide content (A) and skewness (B).

Protein-coding genes and relative synonymous codon usage

The total lengths of the 13 PCGs in the four species range from 11,386 to 11,399 bp, and their GC content varies from 23.67% to 28.65%. The ATN codons were used as the initiation codons for all 13 PCGs. The genes ND1, COX1, COX2, ATP8, ATP6, COX3, ND4L, ND4, ND6, and Cyt-b all start with the ATG codon. The alternative start codon ATT was used for ND2, while the alternative start codon ATA was used for ND3, except in *T. minshanica* (Table S6). In *T. minshanica*, the start codons for ND2 and ND3 of were ATC and ATT, respectively. All of the PCGs adopted AGA、AGG、TAA、TAG or truncated codons T-- or TA- as stop codons. We investigated the codon usage patterns in the mitogenomes within the genus *Tamiops* by RSCU analysis (Fig. S6). The RSCUs of these four mitogenomes exhibited highly similar codon usage patterns. Excluding stop codons, there are a total of 3,789 codons present in the mitogenomes of these species. Furthermore, the most frequently used amino acid was Serine (Ser) and Leucine (Leu), followed by Threonine (Trp) and Phenylalanine (Phe). Additionally, the most commonly used codons were UCA (Val), CGA (Arg), GGA (Gly), and UGA (Trp).


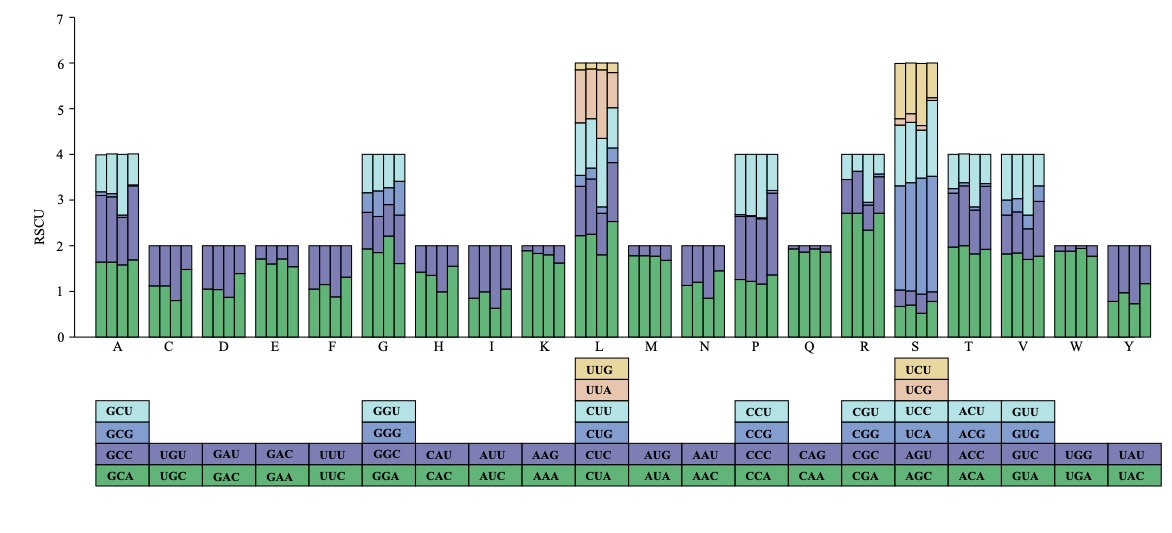


Figure S6. Relative synonymous codon usage (RSCU) of mitochondrial PCGs in four species of the genus *Tamiops*. The proportion of each amino acid used in the construction of the 13 PCGs is displayed at the top of the bar graph. From left to right, the species represented are *T. swinhoei*, *T. maritimus*, *T. mcclellandii*, and *T. minshanica*.

Transfer RNA and ribosomal RNA

The sizes of 22 tRNA genes across four species of the genus *Tamiops* ranged from 61 to 73 bp, with the total size for four species varying from 1,518 to 1,521 bp (Table S6). The G+C content of the tRNAs was higher than that of the A+T content, and both the GC-skew and AT-skew of the tRNAs among the four *Tamiops* species were positive. The secondary structure of tRNAs typically consists of canonical cloverleaf structures, which include the amino acid acceptor arm, the TΨC arm, the variable loop, the anticodon (AC) stem, and the dihydrouridine (DHU) arm. Among the 22 tRNAs in all species of the genus *Tamiops*, 21 tRNAs exhibited a complete secondary structure, with the exception of trnS1(AGN). The trnS1(AGN) displayed a broken secondary structure, lacking the D-arm, a phenomenon commonly observed in metazoan mitogenomes (Fig. S7). Some tRNA bases exhibited mismatches, such as AA pairing and UU pairing, leading to the formation of small loops. Four species shared the same positions of two rRNAs: 12S rRNA and 16S rRNA. The 12S rRNA (rrnS) was located between *tRNA ^phe^* and *tRNA ^Val^*, while the 16S rRNA (rrnL) was situated between *tRNA ^Val^* and *tRNA ^Leu^* (Table S6, Fig. S7). The longest non-coding region is the control region (D-loop), which is located between the *tRNA ^pro^* and *tRNA ^phe^* genes in the mitogenomes of the genus *Tamiops*. Its length ranged 1,043 bp to 1,095 bp. The OL region, which is the origin of light strand replication, was situated between *tRNA ^Asn^* and *tRNA ^Cys^* genes, measuring 29 or 30 bp in length for the four species. The OL region undergoes spatial structural folding to form a stem-loop structure.


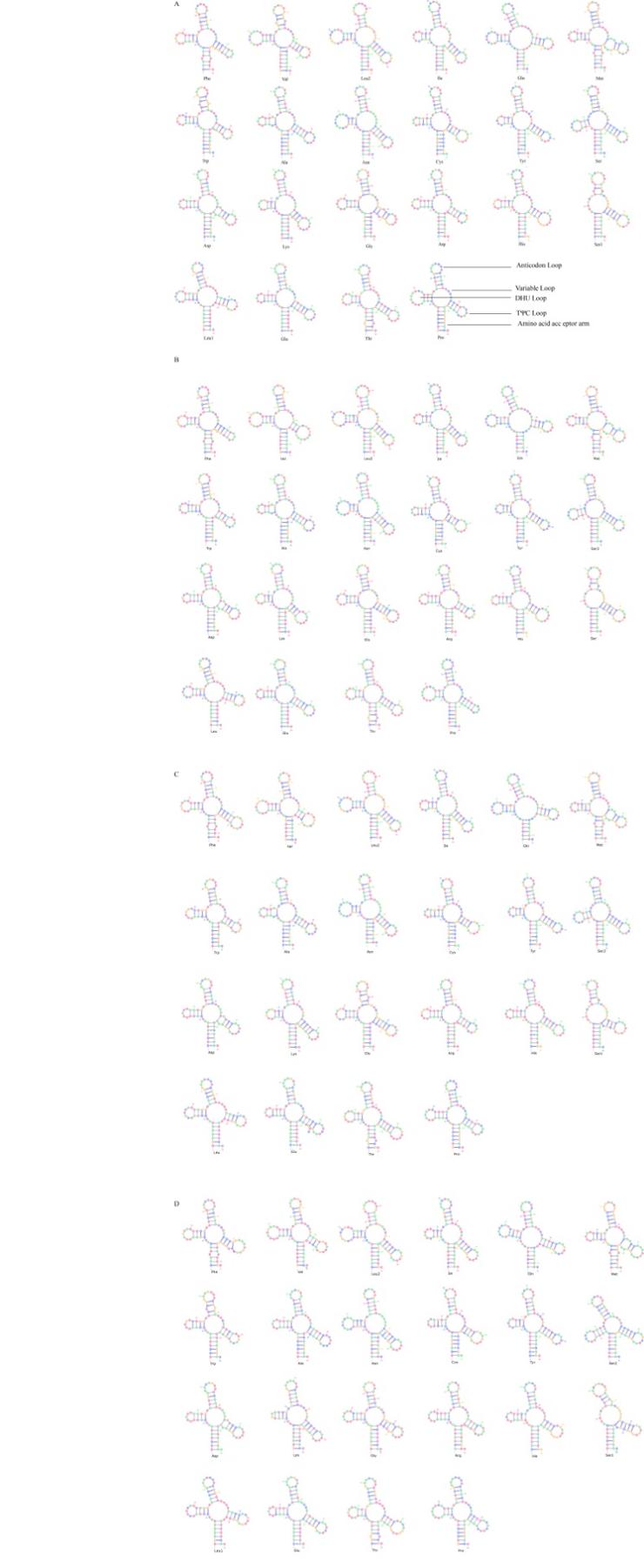


Figure S7. Secondary structures from the 22 tRNAs genes of the genus *Tamiops*. The structures of tRNAs genes are presented in the following order: A) *T. swinhoei*, B) *T. maririmus*, C) *T. mcclellandii*, and D) *T. minshanica*.

Ka/ks calculating

The mitogenomes of *T. rodolphii* was not currently available for this study. The evolutionary patterns of the 13 PCGs across seven clades of the mitochondrial tree within the genus *Tamiops* were analyzed using the Ka/Ks ratios (Fig. S8). All 13 PCGs exhibited low Ka/Ks ratios, with values less than 1, which suggesting that purifying selection was likely the main driver of mitochondrial PCG evolution. The genes COX1 and COX2 had the lowest average Ka/Ks ratios, at 0.008 and 0.011, respectively. Similarly, the genes ATP6, COX3, ND4L, and Cyt-b displayed relatively low Ka/Ks ratios (Fig. 6). The gene ATP8 had the highest mean Ka/Ks ratio, at 0.103.


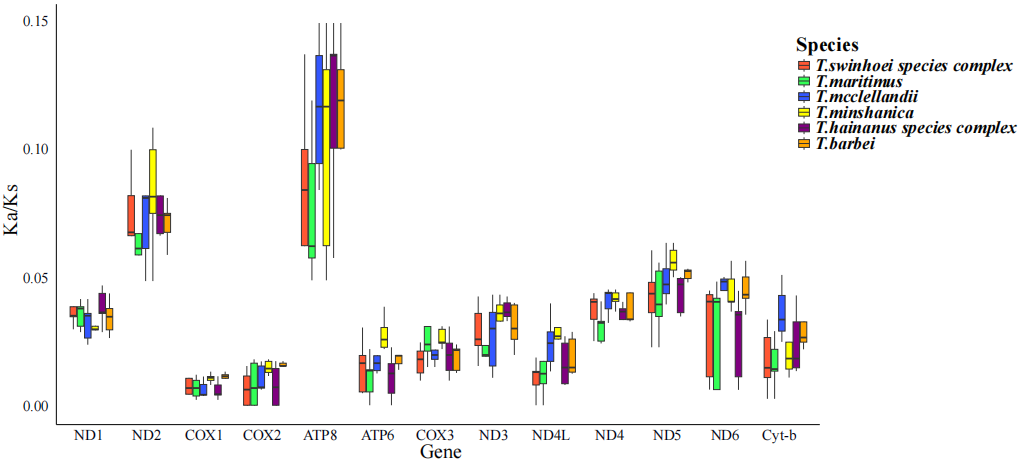


Figure S8. The pairwise nonsynonymous and synonymous ratio (Ka/Ks) of 13 PCGs across six species within the genus *Tamiops.*

Discussion

This study shed light on the mitogenomic characterization, phylogenetics and taxonomy of Asian striped squirrels through comparative mitogenomic and evolutionary analyses. The mitogenomes of four species within the genus *Tamiops* exhibit similarities in length, gene arrangement, and base composition, with no gene rearrangements detected. The variations in length among these mitogenomes are primarily attributed to the control regions, which are commonly found in Sciuridae species (Boukhdoud et al. 2021; Ryu et al. 2013). The complete mitogenomes of the genus *Tamiops* contain 13 PCGs, 22 tRNAs, 2 rRNAs, and two non-coding regions (Table S6), consistent with the canonical arrangement observed in rodents (Horner et al. 2007). Intergenic spaces and overlapping regions between genes have also been observed in *Anaxyrus americanus* and *Boleophthalmus dussumieri* (Cai et al. 2020; Muhala et al. 2024). The most significant overlap among coding genes is occurs between ATP6 and ATP8, spanning 43 bp (Table S6), which aligns with similar phenomena observed in *Dremomys rufigenis* and *Petaurista hainana* within the Sciuridae family (Wang, 2013). In terms of base composition, the mitogenomes of the genus *Tamiops* were biased toward A and T (Fig. S5), a characteristic shared with other vertebrates, such as *Phrynocephalus helioscopus* (Wu et al. 2022). The positive AT skewness, indicating a higher usage of A compared to T, is a compositional pattern also observed in the mitogenome of *Bos indicus* (Arya et al. 2024). Variability in start codons and incomplete stop codons for PCGs has also been reported in other rodent mitochondrial genomes (Table S6) (Petrova et al. 2024; Song et al. 2020). The PCGs frequently initiate with unusual codons, such as ATT, a phenomenon that has also been documented in two toad species (Cai et al. 2020).

RSCU calculations indicate a similar usage pattern across *Tamiops* taxa. The codons CUA, CGA, and UCA, which correspond to Lysine (Lys), Arginine (Arg), and Serine (Ser), respectively, exhibited RSCU values greater than 2 and constituted a higher percentage in the mitogenomes (Fig. S6). The RSCU analysis reveals a higher frequency of A and T nucleotides at the third codon position compared to G and C nucleotides, a pattern also noted in other rodent species, including *Microtus fortis calamorum* (Jiang et al. 2012). The arrangement of all tRNA genes exhibited the typical clover-leaf structure, with the exception of trnS1 (Fig. S7). The tRNA S1 lacks the DHU arm; however, this secondary structure pattern is characteristic of mammals (Arya et al. 2024; Ding et al. 2016; Garey et al. 1989; Yu et al. 2022). The Ka/Ks ratios of all the PCGs were less than 1(Fig. S8), indicating that they have evolved under purifying selection and likely maintain a high level of functional conservation throughout evolution (Hassanin et al. 2005; Hurst, 2002). Among the 13 PCGs, ATP8 exhibited the highest Ka/Ks value, suggesting greater amino acid variability in this biomolecule. The ATP8 gene may have evolved more rapidly than the other PCGs due to slight selective pressure (Hassanin et al. 2005).

Reference

Arya M, Ghosh A, Tyagi K, Tyagi I, Bisht SS, Kumar V (2024) Characterization of Complete Mitochondrial Genome of Badri Breed of *Bos indicus* (Bovidae: Bovinae): Selection Pressure and Comparative Analysis. Biochemical genetics 63(1): 43–66.

Boukhdoud L, Parker LD, Mcinerney NR, Saliba C, Kahale R, Cross H, Matisoo-Smith E, Maldonado JE, Bou Dagher Kharrat M (2021) First mitochondrial genome of the Caucasian squirrel *Sciurus anomalus* (Rodentia, Sciuridae). Mitochondrial DNA B Resour 6(3): 883–885.

Cai YT, Li Q, Zhang JY, Storey KB, Yu DN (2020) Characterization of the mitochondrial genomes of two toads, *Anaxyrus americanus* (Anura: Bufonidae) and *Bufotes pewzowi* (Anura: Bufonidae), with phylogenetic and selection pressure analyses. PeerJ 8: e8901.

Ding L, Li W, Liao J (2016) Mitochondrial genome of *Cricetulus migratorius* (Rodentia: Cricetidae): Insights into the characteristics of the mitochondrial genome and the phylogenetic relationships of *Cricetulus* species. Gene 595(1): 121–129.

Garey JR, Wolstenholme DR (1989) Platyhelminth mitochondrial DNA: evidence for early evolutionary origin of a tRNA(serAGN) that contains a dihydrouridine arm replacement loop, and of serine-specifying AGA and AGG codons. Journal of molecular evolution 28(5): 374–387.

Hassanin A, Léger N, Deutsch J (2005) Evidence for multiple reversals of asymmetric mutational constraints during the evolution of the mitochondrial genome of metazoa, and consequences for phylogenetic inferences. Systematic biology 54(2): 277–298.

Horner DS, Lefkimmiatis K, Reyes A, Gissi C, Saccone C, Pesole G (2007) Phylogenetic analyses of complete mitochondrial genome sequences suggest a basal divergence of the enigmatic rodent *Anomalurus*. BMC evolutionary biology 7: 16.

Hurst LD (2002) The Ka/Ks ratio: Diagnosing the form of sequence evolution. Trends in Genetics: TIG 18(9): 486–487.

Jiang X, Gao J, Ni L, Hu J, Li K, Sun F, Xie J, Bo X, Gao C, Xiao J, Zhou Y (2012) The complete mitochondrial genome of *Microtus fortis calamorum* (Arvicolinae, Rodentia) and its phylogenetic analysis. Gene 498(2): 288–295.

Muhala V, Guimarães-Costa A, Bessa-Silva AR, Rabelo LP, Carneiro J, Macate IE, Watanabe L, Balcázar OD, Gomes GE, Vallinoto M, Sampaio I (2024) Comparative mitochondrial genome brings insights to slight variation in gene proportion and large intergenic spacer and phylogenetic relationship of mudskipper species. Scientific reports 14(1): 3358.

Petrova TV, Panitsina VA, Bodrov SY, Abramson NI (2024) The mitochondrial genome of the critically endangered enigmatic Kazakhstani endemic *Selevinia betpakdalaensis* (Rodentia: Gliridae) and its phylogenetic relationships with other dormouse species. Scientific reports 14(1): 22259.

Ryu SH, Kwak MJ, Hwang UW (2013) Complete mitochondrial genome of the Eurasian flying squirrel *Pteromys volans* (Sciuromorpha, Sciuridae) and revision of rodent phylogeny. Molecular biology reports 40(2): 1917–1926.

Song P, Gao H, Jiang F, Zhang T, Cai Z (2020) Characteristics of the mitochondrial genome of Qinling zokor (*Eospalax rufescens*). Mitochondrial DNA. Part B, Resources 5(3): 2161–2162.

Wang YL (2013) Molecular taxonomic redefinition of three species of Sciuridae in Hainan Island and their characteristics of mitochondrial genome. Master's thesis, Shandong University.

Wu N, Liu J, Wang S, Guo X (2022) Comparative Analysis of Mitochondrial Genomes in Two Subspecies of the Sunwatcher Toad-Headed Agama (Phrynocephalus helioscopus): Prevalent Intraspecific Gene Rearrangements in Phrynocephalus. Genes 13(2): 203.

Yu T, Zhang Y, Zheng WQ, Wu S, Li G, Zhang Y, Li N, Yao R, Fang P, Wang J, Zhou XL (2022) Selective degradation of tRNASer (AGY) is the primary driver for mitochondrial seryl-tRNA synthetase-related disease. Nucleic acids research 50(20): 11755–11774.
