# Supplementary material for: Multilocus Phylogeny of Asiatic Striped Squirrels (Sciuridae, Tamiops) Reveals Their Evolutionary Relationships and Species Limits
Source: Ecol Evol. 2026 Feb 11;16(2):e73099. doi: 10.1002/ece3.73099 (PMC12893811; doi:10.1002/ece3.73099)
Supplement: Supplementary file 1 — Data S1: ece373099‐sup‐0001‐DataS1.zip. Appendix S1:. Results and discussion of comparative mitochondrial genomes analysis in this study. Figure S1: Maximum likelihood phylogenetic trees of the genus Tamiops constructed using various datasets. A. maximum likelihood phylogenetic tree based on the Cyt‐b dataset; B. Maximum likelihood phylogenetic tree based on the Cyt‐b + nuDNA datasets; C. maximum likelihood phylogenetic trees based on nuDNA dataset; D. maximum likelihood phylogenetic trees based on 13PCGs dataset. Figure S2: Photos of the pelage of Tamiops hainanus specimens. Figure S3: Comparative Cranial and Mandibular Characteristics of Tamiops maritimus and Tamiops hainanus. The upper section of the figure, labeled A1–A4, depicts the ventral, dorsal, and lateral views, as well as the mandibular structure of T. maritimus , respectively. The lower section, also labeled A1–A4, shows the ventral, dorsal, and lateral views, along with the mandibular structure of T. hainanus, respectively. Figure S4: Circular maps of the mitogenomes of T. swinhoei (A), T. maritimus (B), T. mcclellandii (C), and T. minshanica (D). Orange blocks represent rRNAs genes, green blocks indicate tRNAs genes, blue blocks denote PCGs, and brownish blocks illustrate the control region and origin of replication. Figure S5: Nucleotide composition of various mitogenome datasets. Hierarchical clustering of Tamiops species (y‐axis) based on nucleotide content (A) and skewness (B). Figure S6: Relative synonymous codon usage (RSCU) of mitochondrial PCGs in four species of the genus Tamiops. The proportion of each amino acid used in the construction of the 13 PCGs is displayed at the top of the bar graph. From left to right, the species represented are T. swinhoei , T. maritimus , T. mcclellandii , and T. minshanica. Figure S7: Secondary structures from the 22 tRNAs genes of the genus Tamiops. The structures of tRNAs genes are presented in the following order: (A) T. swinhoei , (B) T. maririmus, (C) T. [file ECE3-16-e73099-s001.zip › Table_S1.docx]

| Table S1. GenBank accession numbers for the DNA sequences used in the phylogenetic analyses presented in this study. | | | | | | | | | | |
| --- | --- | --- | --- | --- | --- | --- | --- | --- | --- | --- |
| Museum/ GenBank No. | Species | CYTB | Sample localities | IRBP | Sample localities | RAG1 | Sample localities | PRKCI | Sample localities | Reference |
| GenBank | *C. erythraeus* | AB499908 | Tam Dao, Vietnam | HQ698523 | Zhongxingxincun, Nantou, Taiwan | HQ698439 | Zhongxingxincun, Nantou, Taiwan | HQ698411 | Zhongxingxincun, Nantou, Taiwan | Oshida et al. 2001; Chang et al. 2011 |
| GenBank | *C. erythraeus* | / | / | HQ698522 | Kaohsiung, Tianchi, Taiwan | HQ698438 | Kaohsiung, Tianchi, Taiwan | HQ698412 | Shishan, Nantou, Taiwan | Chang et al. 2011 |
| GenBank | *C. inornatus* | AB499905 | Co Ma, Thuan, Chau, Son La, Vietnam | KX192422 | / | / | / | / | / | Oshida et al. 2011; Balakirev. 2016 |
| GenBank | *C. inornatus* | AB499906 | Hon, Phu Yen, Son La, Vietnam | / | / | / | / | / | / | Oshida et al. 2011 |
| GenBank | *C. caniceps* | AB499919 | Pasoh Forest Reserve, Negeri Sembilan, Malaysia | / | / | / | / | / | / | Oshida et al. 2011 |
| GenBank | *Sundasciurus brookei* | KP120729 | Borneo | AY227577 | / | / | / | / | / | Tex et al. 2010; Mercer et al. 2003 |
| GenBank | *S. juvencus* | KP120737 | Palawan, Philippines | */* | */* | */* | */* | */* | */* | den Tex et al. 2010 |
| GenBank | *S. juvencus* | KP120738 | Ikadwang, Banwa Island | */* | */* | */* | */* | */* | */* | den Tex et al. 2010 |
| GenBank | *S. juvencus* | KP120739 | Ikadwang, Banwa Island | */* | */* | */* | */* | */* | */* | den Tex et al. 2010 |
| GenBank | *S. philippinensis* | KP120745 | Mindanao, Philippines | */* | */* | */* | */* | */* | */* | den Tex et al. 2010 |
| GenBank | *S. philippinensis* | KP120746 | Mindanao, Philippines | */* | */* | */* | */* | */* | */* | den Tex et al. 2010 |
| GenBank | *S. samarensis* | KP120748 | / | */* | */* | */* | */* | */* | */* | den Tex et al. 2010 |
| GenBank | *S. samarensis* | KP120749 | Leyte Island | */* | */* | */* | */* | */* | */* | den Tex et al. 2010 |
| GenBank | *S. tenuis* | KP120751 | Malay Peninsula | */* | */* | */* | */* | */* | */* | den Tex et al. 2010 |
| GenBank | *S. tenuis* | KP120752 | Malay Peninsula | */* | */* | */* | */* | */* | */* | den Tex et al. 2010 |
| GenBank | *Dremomys gularis* | EF539337 | / | / | / | / | / | / | / | Li et al. 2008 |
| GenBank | *D. gularis* | EF539338 | / | / | / | / | / | / | / | Li et al. 2008 |
| GenBank | *D. gularis* | EF539339 | Jingdong, Yunnan, China | / | / | / | / | / | / | Li et al. 2008 |
| GenBank | *D. lokriah* | EF539335 | Gongshan, Yunnan, China | / | / | / | / | / | / | Li et al. 2008 |
| GenBank | *D.pernyi* | LC150596 | Hehuanshan, Nantou, Taiwan | HQ698525 | Hehuanshan, Nantou, Taiwan | HQ698413 | Hehuanshan, Nantou, Taiwan | HQ698443 | Yilan, Nan’ao, Taiwan | Oshida et al. 2017;  Chang et al. 2011; |
| GenBank | *D.pernyi* | HQ698361 | Hehuanshan, Nantou, Taiwan | HQ698526 | Kaohsiung, Tengzhi, Taiwan | HQ698414 | Kaohsiung:Tengzhi, Taiwan | HQ698441 | Hehuanshan,  Nantou, Taiwan | Chang et al. 2011; |
| GenBank | *D.pernyi* | HQ698362 | Kaohsiung:Tengzhi, Taiwan | HQ698527 | Yilan, Nan’ao, Taiwan | HQ698415 | Yilan, Nan’ao, Taiwan | HQ698442 | Kaohsiung:Tengzhi, Taiwan | Chang et al. 2011; |
| GenBank | *D.pyrrhomerus* | EF539342 | Pingbian, Yunnan, China | / | / | / | / | / | / | Li et al. 2008 |
| GenBank | *D.pyrrhomerus* | KP708705 | / | / | / | / | / | / | / | Unpublished |
| GenBank | *D. rufigenis* | LC150597 | Tam Dao, Vietnam | KX171260 | / | KX171273 | / | / | / | Oshida et al. 2017; Balakirev. 2016 |
| GenBank | *D. rufigenis* | LC150598 | Tam Dao, Vietnam | KX171259 | / | KX171274 | / | / | / | Oshida et al. 2017; Balakirev. 2016 |
| GenBank | *D. rufigenis* | LC150599 | Tam Dao, Vietnam | KX171254 | / | KX171275 | / | / | / | Oshida et al. 2017; Balakirev. 2016 |
| GenBank | *Tamiops maritimus_FU4* | HQ698367 | Wuyishan, Fujian, China | HQ698528 | Wuyishan, Fujian, China | HQ698444 | Wuyishan, Fujian, China | HQ698416 | Wuyishan, Fujian, China | Chang et al. 2011 |
| GenBank | *T. maritimus_FU5* | HQ698368 | Wuyishan, Fujian, China | HQ698529 | Wuyishan, Fujian, China | HQ698445 | Wuyishan, Fujian, China | HQ698417 | Wuyishan, Fujian, China | Chang et al. 2011 |
| GenBank | *T. maritimus_FU6* | HQ698369 | Wuyishan, Fujian, China | HQ698530 | Wuyishan, Fujian, China | HQ698446 | Wuyishan, Fujian, China | HQ698418 | Wuyishan, Fujian, China | Chang et al. 2011 |
| GenBank | *T. maritimus_TW1* | HQ698370 | Tianchi, Kaohsiung, Taiwan, China | HQ698531 | Tianchi, Kaohsiung, Taiwan, China | HQ698447 | Tianchi, Kaohsiung,Taiwan, China | HQ698419 | Tianchi, kaohsiung, Taiwan, China | Chang et al. 2011 |
| GenBank | *T. maritimus_TW2* | HQ698371 | Taguanshan, kaohsiung, Taiwan, China | HQ698532 | Taguanshan, Kaohsiung,Taiwan,China | HQ698448 | Taguanshan,kaohsiung,Taiwan, China | HQ698420 | Taguanshan,kaohsiung,Taiwan,China | Chang et al. 2011 |
| GenBank | *T. maritimus_TW3* | HQ698372 | Tatajia, Nantou, Taiwan, China | HQ698533 | Tatajia, Nantou, Taiwan, China | HQ698449 | Tatajia, Nantou, Taiwan, China | HQ698421 | Tatajia, Nantou, Taiwan, China | Chang et al. 2011 |
| GenBank | *T. maritimus_TW4* | HQ698373 | Tatajia, Nantou, Taiwan, China | HQ698534 | Tatajia, Nantou, Taiwan, China | HQ698450 | Tatajia, Nantou, Taiwan, China | HQ698422 | Tatajia, Nantou, Taiwan, China | Chang et al. 2011 |
| GenBank | *T. maritimus_TD1* | HQ698378 | Tam Dao, Vinh Phuc, Vietnam | HQ698535 | Tam Dao, Vinh Phuc, Vietnam | HQ698451 | Tam Dao, Vinh Phuc, Vietnam | HQ698423 | Tam Dao, Vinh Phuc, Vietnam | Chang et al. 2011 |
| GenBank | *T. maritimus_TD3* | HQ698380 | Tam Dao, Vinh Phuc, Vietnam | HQ698536 | Tam Dao, Vinh Phuc, Vietnam | HQ698452 | Tam Dao, Vinh Phuc, Vietnam | HQ698424 | Tam Dao, Vinh Phuc, Vietnam | Chang et al. 2011 |
| GenBank | *T. maritimus_TD4* | HQ698381 | Tam Dao, Vinh Phuc, Vietnam | HQ698537 | Tam Dao, Vinh Phuc, Vietnam | HQ698453 | Tam Dao, Vinh Phuc, Vietnam | HQ698425 | Tam Dao, Vinh Phuc, Vietnam | Chang et al. 2011 |
| GenBank | *T. maritimus_PY1* | HQ698382 | Phu Yen, Sonla, Vietnam | HQ698538 | Phu Yen, Sonla, Vietnam | HQ698454 | Phu Yen, Sonla, Vietnam | HQ698426 | Phu Yen, Sonla, Vietnam | Chang et al. 2011 |
| GenBank | *T. maritimus_PY5* | HQ698386 | Phu Yen, Sonla, Vietnam | HQ698539 | Phu Yen, Sonla, Vietnam | HQ698455 | Phu Yen, Sonla, Vietnam | HQ698427 | Phu Yen, Sonla, Vietnam | Chang et al. 2011 |
| GenBank | *T. maritimus_PY6* | HQ698387 | Phu Yen, Sonla, Vietnam | HQ698540 | Phu Yen, Sonla, Vietnam | HQ698456 | Phu Yen, Sonla, Vietnam | HQ698428 | Phu Yen, Sonla, Vietnam | Chang et al. 2011 |
| GenBank | *T. mcclellandii_TL1* | HQ698388 | Cheng Rai, Thailand | HQ698541 | Cheng Rai, Thailand | HQ698457 | Cheng Rai, Thailand | HQ698429 | Cheng Rai, Thailand | Chang et al. 2011 |
| GenBank | *T. mcclellandii_TL3* | HQ698390 | Mae Chan, Thailand | HQ698542 | Mae Chan, Thailand | HQ698458 | Mae Chan, Thailand | HQ698430 | Mae Chan, Thailand | Chang et al. 2011 |
| GenBank | *T. rodolphii_KL2* | HQ698395 | kien Luong, Kien Giang, Vietnam | HQ698543 | kien Luong, kien Giang, Vietnam | HQ698459 | kien Luong, kien Giang, Vietnam | HQ698431 | kien Luong, kien Giang, Vietnam | Chang et al. 2011 |
| GenBank | *T. rodolphii_KL3* | HQ698396 | kien Luong, Kien Giang, Vietnam | HQ698544 | kien Luong, kien Giang, Vietnam | HQ698460 | kien Luong, kien Giang, Vietnam | HQ698432 | kien Luong, kien Giang, Vietnam | Chang et al. 2011 |
| GenBank | *T. rodolphii_KL5* | HQ698398 | kien Luong, Kien Giang, Vietnam | HQ698545 | kien Luong, kien Giang, Vietnam | HQ698461 | kien Luong, kien Giang, Vietnam | HQ698433 | kien Luong, kien Giang, Vietnam | Chang et al. 2011 |
| GenBank | *T. swinhoei_SP1* | HQ698401 | Sapa, Lao Cai, Vietnam | HQ698546 | Sapa, Lao Cai, Vietnam | HQ698462 | Sapa, Lao Cai, Vietnam | HQ698434 | Sapa, Lao Cai, Vietnam | Chang et al. 2011 |
| GenBank | *T. swinhoei_SP2* | HQ698402 | Sapa, Lao Cai, Vietnam | HQ698547 | Sapa, Lao Cai, Vietnam | HQ698463 | Sapa, Lao Cai, Vietnam | HQ698435 | Sapa, Lao Cai, Vietnam | Chang et al. 2011 |
| GenBank | *T. swinhoei_SP3* | HQ698403 | Sapa, Lao Cai, Vietnam | HQ698548 | Sapa, Lao Cai, Vietnam | HQ698464 | Sapa, Lao Cai, Vietnam | HQ698436 | Sapa, Lao Cai, Vietnam | Chang et al. 2011 |
| GenBank | *T. swinhoei_SS* | HQ698409 | Shimian, Sichuan, China | HQ698549 | Shimian, Sichuan, China | HQ698465 | Shimian, Sichuan, China | HQ698437 | Shimian, Sichuan, China | Chang et al. 2011 |
| GenBank | *Tamiops minshanica* | OK432502 | Wanglang, Sichuan, China | OK432506 | Wanglang, Sichuan, China | OK432509 | Wanglang, Sichuan, China | OK432512 | Wanglang, Sichuan, China | Liu et al.2022 |
| GenBank | *T. minshanica* | OK432501 | Wanglang, Sichuan, China | OK432505 | Wanglang, Sichuan, China | OK432508 | Wanglang, Sichuan, China | OK432511 | Wanglang, Sichuan, China | Liu et al.2022 |
| GenBank | *T. minshanica* | OK432499 | Wanglang, Sichuan, China | OK432503 | Wanglang, Sichuan, China | OK432507 | Wanglang, Sichuan, China | OK432510 | Wanglang, Sichuan, China | Liu et al.2022,  In this study |
| GenBank | *T. s. swinhoei* | OK493503 | Baoxing, Sichuan, China | OK493505 | Baoxing, Sichuan, China | OK493507 | Baoxing, Sichuan, China | OK493509 | Baoxing, Sichuan, China | Liu et al.202 |
|  |  |  |  |  |  |  |  |  |  |  |
| SAF201148 | *Tamiops maritimus hainanus* | PQ591913 | Hainan, China | / | Hainan, China | PQ629581 | Hainan, China | PQ629564 | Hainan, China | In this study |
| CSD7748 | *T. m. hainanus* | PQ591914 | Hainan, China | PQ629549 | Hainan, China | PQ629582 | Hainan, China | PQ629565 | Hainan, China | In this study |
| CSD6956 | *Tamiops mcclellandii mcclellandii* | PQ591912 | Motuo, Xizang, China | PQ629548 | Motuo, Xizang, China | PQ629580 | Motuo, Xizang, China | PQ629563 | Motuo, Xizang, China | In this study |
| SAF181596 | *Tamiops minshanica* | OK432500 | Wanglang, Sichuan, China | OK432504 | Wanglang, Sichuan, China | / | / | / | / | Liu et al.2022,  In this study |
| SAF19631 | *T. s. swinhoei* | PQ629532 | Pingwu, Sichuan, China | PQ629541 | Pingwu, Sichuan, China | PQ629573 | Pingwu, Sichuan, China | PQ629556 | Pingwu, Sichuan, China | In this study |
| SAF19632 | *T. s. swinhoei* | PQ629533 | Pingwu, Sichuan, China | PQ629542 | Pingwu, Sichuan, China | PQ629574 | Pingwu, Sichuan, China | PQ629557 | Pingwu, Sichuan, China | In this study |
| SAF19730 | *T. s. swinhoei* | PQ629534 | Pingwu, Sichuan, China | PQ629543 | Pingwu, Sichuan, China | PQ629575 | Pingwu, Sichuan, China | PQ629558 | Pingwu, Sichuan, China | In this study |
| SAF19765 | *T. s. swinhoei* | PQ629535 | Pingwu, Sichuan, China | PQ629544 | Pingwu, Sichuan, China | PQ629576 | Pingwu, Sichuan, China | PQ629559 | Pingwu, Sichuan, China | In this study |
| SAF19766 | *T. s. swinhoei* | PQ629536 | Pingwu, Sichuan, China | PQ629545 | Pingwu, Sichuan, China | PQ629577 | Pingwu, Sichuan, China | PQ629560 | Pingwu, Sichuan, China | In this study |
| SAF09282 | *T. s. swinhoei* | OK493504 | Baoxing, Sichuan, China | OK493506 | Baoxing, Sichuan, China | OK493508 | Baoxing, Sichuan, China | OK493510 | Baoxing, Sichuan, China | Liu et al.202,  In this study |
| CSD5270 | *T. s. swinhoei* | PQ629537 | Emeishan, Sichuan, China | PQ629546 | Emeishan, Sichuan, China | PQ629578 | Emeishan, Sichuan, China | PQ629561 | Emeishan, Sichuan, China | In this study |
| CSD6395 | *T. s. swinhoei* | PQ591911 | Emeishan, Sichuan, China | PQ629547 | Emeishan, Sichuan, China | PQ629579 | Emeishan, Sichuan, China | PQ629562 | Emeishan, Sichuan, China | In this study |
| SAF11622 | *T. s. swinhoei* | PQ629529 | Xiaojin, Sichuan, China | PQ629538 | Xiaojin, Sichuan, China | PQ629570 | Xiaojin, Sichuan, China | PQ629553 | Xiaojin, Sichuan, China | In this study |
| SAF16601 | *T. s. swinhoei* | PQ629530 | Minya Konka, Sichuan, China | PQ629539 | Minya Konka, Sichuan, China | PQ629571 | Minya Konka, Sichuan, China | PQ629554 | Minya Konka, Sichuan, China | In this study |
| SAF05486 | *T. s. swinhoei* | PQ629531 | Danba, Sichuan, China | PQ629540 | Danba, Sichuan, China | PQ629572 | Danba, Sichuan, China | PQ629555 | Danba, Sichuan, China | In this study |
| SAF14745 | *T. s. clarkei* | PQ591915 | Derong, Sichuan, China | / | Derong, Sichuan, China | PQ629583 | Derong, Sichuan, China | PQ629566 | Derong, Sichuan, China | In this study |
| SAF15254 | *T. s. russeolus* | PQ591920 | Deqin, Yunnan, China | PQ629552 | Deqin, Yunnan, China | PQ629586 | Deqin, Yunnan, China | PQ629569 | Deqin, Yunnan, China | In this study |
| SAF19814 | *T. s. forresti* | PQ591918 | Lijiang, Yunnan, China | PQ629551 | Lijiang, Yunnan, China | PQ629585 | Lijiang, Yunnan, China | PQ629568 | Lijiang, Yunnan, China | In this study |
| SAF13049 | *T. maritimus* | PQ591916 | Tongren, Guizhou, China | PQ629550 | Tongren, Guizhou, China | PQ629584 | Tongren, Guizhou, China | PQ629567 | Tongren, Guizhou, China | In this study |

References:

Balakirev, A.E., 2016. Phylogeography and taxonomy of Asian red-cheeked squirrels (Rodentia, Sciuridae, *Dremomys*) in Vietnam. Zootaxa.

Chang, S.W., Oshida, T., Endo, H., Nguyen, S.T., Dang, C.N., Nguyen, D.X., et al., 2011. Ancient hybridization and underestimated species diversity in Asian striped squirrels (genus *Tamiops*): inference from paternal, maternal and biparental markers. Journal of Zoology. 285, 128–138.

Tex, R.J., Thorington, R., Maldonado, J.E., Leonard, J.A., 2010. Speciation dynamics in the SE Asian tropics: Putting a time perspective on the phylogeny and biogeography of Sundaland tree squirrels, *Sundasciurus*. Molecular phylogenetics and evolution. 55(2), 711–720.

Song, L., Yu, F., Su, Y., Wang, Y., Jiang, X., Mcguire, P.M., et al., 2008. Molecular phylogeny of five species of *Dremomys* (Rodentia: Sciuridae), inferred from cytochrome b gene sequences. Zoologica Scripta. 37(4), 349-354.

Mercer, J.M., Roth, V.L., 2003. The effects of Cenozoic global change on squirrel phylogeny. Science. 299, 1568-1572.

Oshida, T., Dang, C.N., Nguyen, S.T., Nguyen, N.X., Hayashi, Y., 2011. Phylogenetic relationship between *Callosciurus caniceps* and *C. inornatus* (Rodentia, Sciuridae): implications for zoogeographical isolation by the Mekong River. Italian Journal of Zoology. 78, 328-335.

Oshida, T., Lin, L.K., Chang, S.W., Dang, C.N., Nguyen, S.T., Nguyen, N.X., et al., 2017. Mitochondrial DNA evidence reveals genetic difference between Perny's long-nosed squirrels in Taiwan and Asian mainland. Mammal Study. 42, 111-116.
