# Supplementary material for: Multilocus Phylogeny of Asiatic Striped Squirrels (Sciuridae, Tamiops) Reveals Their Evolutionary Relationships and Species Limits
Source: Ecol Evol. 2026 Feb 11;16(2):e73099. doi: 10.1002/ece3.73099 (PMC12893811; doi:10.1002/ece3.73099)
Supplement: Supplementary file 1 — Data S1: ece373099‐sup‐0001‐DataS1.zip. Appendix S1:. Results and discussion of comparative mitochondrial genomes analysis in this study. Figure S1: Maximum likelihood phylogenetic trees of the genus Tamiops constructed using various datasets. A. maximum likelihood phylogenetic tree based on the Cyt‐b dataset; B. Maximum likelihood phylogenetic tree based on the Cyt‐b + nuDNA datasets; C. maximum likelihood phylogenetic trees based on nuDNA dataset; D. maximum likelihood phylogenetic trees based on 13PCGs dataset. Figure S2: Photos of the pelage of Tamiops hainanus specimens. Figure S3: Comparative Cranial and Mandibular Characteristics of Tamiops maritimus and Tamiops hainanus. The upper section of the figure, labeled A1–A4, depicts the ventral, dorsal, and lateral views, as well as the mandibular structure of T. maritimus , respectively. The lower section, also labeled A1–A4, shows the ventral, dorsal, and lateral views, along with the mandibular structure of T. hainanus, respectively. Figure S4: Circular maps of the mitogenomes of T. swinhoei (A), T. maritimus (B), T. mcclellandii (C), and T. minshanica (D). Orange blocks represent rRNAs genes, green blocks indicate tRNAs genes, blue blocks denote PCGs, and brownish blocks illustrate the control region and origin of replication. Figure S5: Nucleotide composition of various mitogenome datasets. Hierarchical clustering of Tamiops species (y‐axis) based on nucleotide content (A) and skewness (B). Figure S6: Relative synonymous codon usage (RSCU) of mitochondrial PCGs in four species of the genus Tamiops. The proportion of each amino acid used in the construction of the 13 PCGs is displayed at the top of the bar graph. From left to right, the species represented are T. swinhoei , T. maritimus , T. mcclellandii , and T. minshanica. Figure S7: Secondary structures from the 22 tRNAs genes of the genus Tamiops. The structures of tRNAs genes are presented in the following order: (A) T. swinhoei , (B) T. maririmus, (C) T. [file ECE3-16-e73099-s001.zip › Table_S3.docx]

| Table S3. Sequence lengths of 13 PCGs and the best substitution models employed for phylogenetic tree reconstruction. | | |
| --- | --- | --- |
| Partitions | Sequence length | best model |
| ND1 | 957 | HKY+I+G |
| ND2 | 1044 | GTR+I+G |
| COX1 | 1542 | GTR+I+G |
| COX2 | 684 | HKY+I |
| ATP8 | 204 | HKY+G |
| ATP6 | 681 | HKY+I+G |
| COX3 | 785 | HKY+I+G |
| ND3 | 348 | HKY+G |
| ND4L | 297 | HKY+G |
| ND4 | 1378 | HKY+I+G |
| ND5 | 1818 | GTR+G |
| ND6 | 525 | GTR+I+G |
| Cyt-b | 1140 | GTR+I+G |
